# Type I IFN drives neutrophil swarming, impeding lung T cell–macrophage interactions and TB control

**DOI:** 10.1084/jem.20250466

**Published:** 2025-09-23

**Authors:** William J. Branchett, Evangelos Stavropoulos, Jessica Shields, Alaa Al-Dibouni, Marcos Cardoso, Ana Isabel Fernandes, Lúcia Moreira-Teixeira, Hubert Slawinski, Anna Mikolajczak, Angela Rodgers, Margarida Saraiva, Anne O’Garra

**Affiliations:** 1 Immunoregulation and Infection Laboratory, The Francis Crick Institute, London, UK; 2 Immunoregulation Laboratory, i3S, Porto, Portugal; 3 Instituto de Ciências Biomédicas Abel Salazar, University of Porto, Porto, Portugal; 4 Genomics Science Technology Platform, The Francis Crick Institute, London, UK; 5 Experimental Histopathology Science Technology Platform, The Francis Crick Institute, London, UK; 6 Host-Pathogen Interactions in Tuberculosis Laboratory, The Francis Crick Institute, London, UK

## Abstract

The early immune mechanisms determining *Mycobacterium tuberculosis* infection outcome are unclear. Using bulk and scRNA-seq over the first weeks of infection, we describe an unexpected, higher early pulmonary type I IFN response in relatively resistant C57BL/6 as compared with highly TB-susceptible C3HeB/FeJ mice. C57BL/6 mice showed pronounced early monocyte-derived macrophage (MDM) accumulation and extensive CD4^+^ T cell–MDM interactions in lung lesions, accompanied by high expression of T cell–attractant chemokines by MDMs. Conversely, lesions in C3HeB/FeJ mice were dominated by neutrophils with high expression of pro-inflammatory chemokines, from which CD4^+^ T cells were spatially segregated. Early type I IFN signaling blockade reduced bacterial load and neutrophil swarming within early TB lesions while increasing CD4^+^ T cell numbers in both C57BL/6 and C3HeB/FeJ mice, with later more pronounced effects on bacterial load in C3HeB/FeJ mice. These data suggest that early type I IFN signaling during *M. tuberculosis* infection favors neutrophil accumulation and limits CD4^+^ T cell infiltration into developing lesions.

## Teaser

The early immune mechanisms determining *M. tuberculosis* infection outcome are unclear. Type I signaling early during *M. tuberculosis* infection favors neutrophil swarming and limits CD4^+^ T cell–macrophage interactions in TB lesions, impeding TB disease control.

## Introduction

Tuberculosis (TB) contributes to over a million deaths annually (Geneva: World Health Organization, 2024), yet only a small minority of individuals infected with *Mycobacterium tuberculosis* progress to active TB, mostly doing so within 2 years of infection (Behr et al., 2024). This highlights the importance of understanding the host immune response resulting in protection or progression to TB disease. Despite this, the mechanisms of optimal initiation, localization, and regulation of the immune response to *M. tuberculosis* are not well understood (Bloom, 2023; Cohen et al., 2022; O’Garra et al., 2013).

Mouse models form an important part of TB research, allowing experimental manipulation of cells and pathways in an intact mammalian system at considerably higher throughput and lower cost than in nonhuman primates. Most inbred laboratory mouse strains, including C57BL/6, are relatively TB resistant, controlling *M. tuberculosis* infection for several months and failing to develop the necrotic lung lesions observed in human TB (Flynn, 2006; Fortin et al., 2007). However, genetic or pharmacological perturbations on this relatively resistant background of mice have been instrumental in identifying pathways and cells essential for protection against *M. tuberculosis*, including IL-12, IFN-γ, CD4^+^ T cells, and TNF-α (Cooper and Khader, 2008; Flynn, 2006; Fortin et al., 2007; Kramnik and Beamer, 2016), the importance of which have all subsequently been verified as protective in humans (Fortin et al., 2007; Keane et al., 2001).

Some laboratory mouse strains demonstrate variable, genetically determined susceptibility to TB (Kramnik and Beamer, 2016; Meade and Smith, 2025). The TB-susceptible C3HeB/FeJ strain develops high lung bacterial burdens and necrotic lesions with progressive disease resembling human TB pathology (Irwin et al., 2015; Kramnik et al., 2000). Intense research activity was triggered upon identification of a neutrophil-driven type I IFN–inducible signature in the whole blood of active TB patients (Berry et al., 2010; O’Garra et al., 2013), which we have recently reported to be recapitulated in the blood of *M. tuberculosis*–infected C3HeB/FeJ mice (Moreira-Teixeira et al., 2020b). We have subsequently shown that sustained type I IFN signaling resulted in increased bacterial growth and disease severity in these highly TB-susceptible mice (Moreira-Teixeira et al., 2020a). C57BL/6 mice bearing a susceptibility locus from C3HeB/FeJ mice also displayed type I IFN–dependent increases in lung pathology and bacterial loads (Ji et al., 2019), as did mice with a knockout targeting the *Sp140* gene, which lies within this locus (Ji et al., 2021). A further report demonstrated that recruited monocyte-derived macrophages (MDMs) are both major producers of—and responders to—type I IFNs in *Sp140*^−/−^ C57BL/6 mice, at a time point by which extensive lung pathology had developed (Kotov et al., 2023). In contrast, little to no effect of IFNαβ receptor (IFNAR) deletion in wild-type C57BL/6 mice has been reported during aerosol *M. tuberculosis* infection (Desvignes et al., 2012; Ji et al., 2019; Mayer-Barber et al., 2011; Moreira-Teixeira et al., 2017; Moreira-Teixeira et al., 2016; Ordway et al., 2007).

The neutrophil-driven type I IFN–dependent blood signature, which correlates with disease severity in human TB (Berry et al., 2010), along with the observation of increased airway neutrophils in more advanced TB (Condos et al., 1998), have implicated both type I IFN and neutrophils in TB pathogenesis. Moreover, pulmonary neutrophilic inflammation is observed during established TB in multiple susceptible mouse models, in which disease can be ameliorated by neutrophil depletion (Keller et al., 2006; Kimmey et al., 2015; Moreira-Teixeira et al., 2020a; Nandi and Behar, 2011), consistent with a pathogenic role for neutrophils in the context of failed immune control of *M. tuberculosis*. Neutrophils are highly permissive to *M. tuberculosis* replication (Lovewell et al., 2021) and are the most abundant infected cells in respiratory samples from active human TB (Eum et al., 2010). However, the mechanisms by which neutrophils promote TB pathogenesis over the course of infection are incompletely understood. Type I IFN-induced neutrophil extracellular trap (NET) formation in vivo in *M. tuberculosis*–infected C3HeB/FeJ mice (Moreira-Teixeira et al., 2020a) and type I IFN–induced NET release induction of *M. tuberculosis* replication in neutrophils in vitro (Sur Chowdhury et al., 2024) have been reported. However, whether type I IFN more broadly affects neutrophil recruitment, activation, and cell–cell interactions or spatial organization in TB lesions is unclear.

We report here using bulk and single-cell RNA-sequencing (scRNA-seq) that C57BL/6 mice unexpectedly display higher expression of type I IFN–inducible genes in lungs as compared with highly susceptible C3HeB/FeJ mice at early time points after *M. tuberculosis* infection. This was accompanied by increased early bacterial loads and lesion formation in infected C57BL/6 mice, prior to the accumulation of large numbers of effector CD4^+^ T cells in lung lesions and their eventual superior control of infection. Analyses of scRNA-seq data revealed increased early numbers of MDMs and *Ifng*-expressing CD4^+^ T cells in lungs of *M. tuberculosis*–infected C57BL/6 mice, whereas C3HeB/FeJ mice had delayed MDM accumulation accompanied by large numbers of inflammatory neutrophils. Using multiplex immunofluorescence, we identify an inverse relationship between CD4^+^ T cells and neutrophils within lung lesions that is dynamic over the first weeks of infection and dependent on early type I IFN signaling in both C57BL/6 and C3HeB/FeJ mice. Suppression of type I IFN–dependent neutrophil responses facilitated CD4^+^ T cell accumulation within TB lesions, with type I IFN signaling impeding early *M. tuberculosis* control in both mouse strains. This study provides a valuable resource for understanding the establishment of protective and failed immune responses to *M. tuberculosis* and highlights the importance of understanding neutrophil and T cell dynamics and the effects of type I IFN on neutrophils and macrophage–CD4^+^ T cell interactions in the lung at early stages of infection.

Bulk and scRNA-seq gene expression datasets presented here on potential pathways of protection and pathogenesis in experimental mouse TB models are easily accessible as a resource using an online web app: https://ogarra.shinyapps.io/earlymousetb/.

## Results

### Earlier immune response following *M. tuberculosis* infection in C57BL/6 compared with TB-susceptible C3HeB/FeJ mice

**Figure 1.**
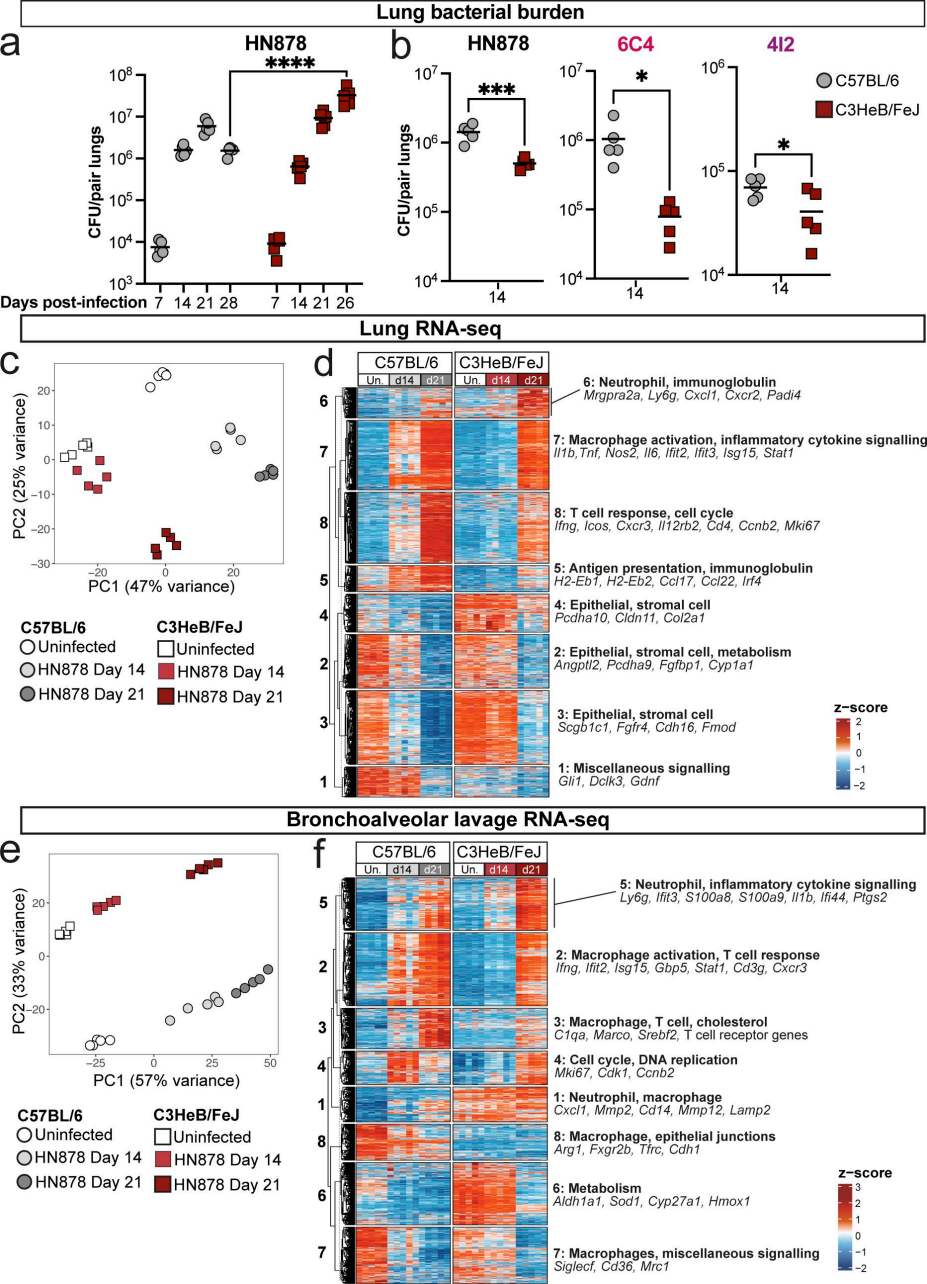
**Increased *M. tuberculosis* load and earlier immune response in C57BL/6 as compared with TB-susceptible C3HeB/FeJ-infected mice. (a and b)** C57BL/6 and C3HeB/FeJ mice were aerosol infected with the indicated *M. tuberculosis* strains, and *M. tuberculosis* CFUs in lung tissue were determined at the indicated time points. Data in a and b are from single experiments with *N* = 5 mice per group and are representative of a minimum of two independent experiments. Statistical analysis: (a) two-way ANOVA with Holm–Sidak post hoc test; (b) unpaired *t* test for HN878 and 6C4, unpaired *t* test with Welch’s correction for 4I2; *, P < 0.05; ***, P < 0.001; ****, P < 0.0001. **(c–f)** Bulk RNA-seq was performed on whole lung tissue and whole BAL cell pellets from mice infected with HN878 at the indicated time points, as compared with uninfected controls. **(c and e)** Principal component analysis of all protein-coding, immunoglobulin, and T cell–receptor genes in bulk RNA-seq data from whole lung tissue and whole BAL cell pellets. **(d and f)** All DEGs in whole lung and BAL at any time point compared with the respective uninfected controls were subjected to k-means clustering. Clusters are annotated based on representative hallmark genes and pathways. Data in c–f are from a single bulk RNA-seq experiment with *N* = 5 mice per group. See also Fig. S1, a and b.

**Figure S1.**
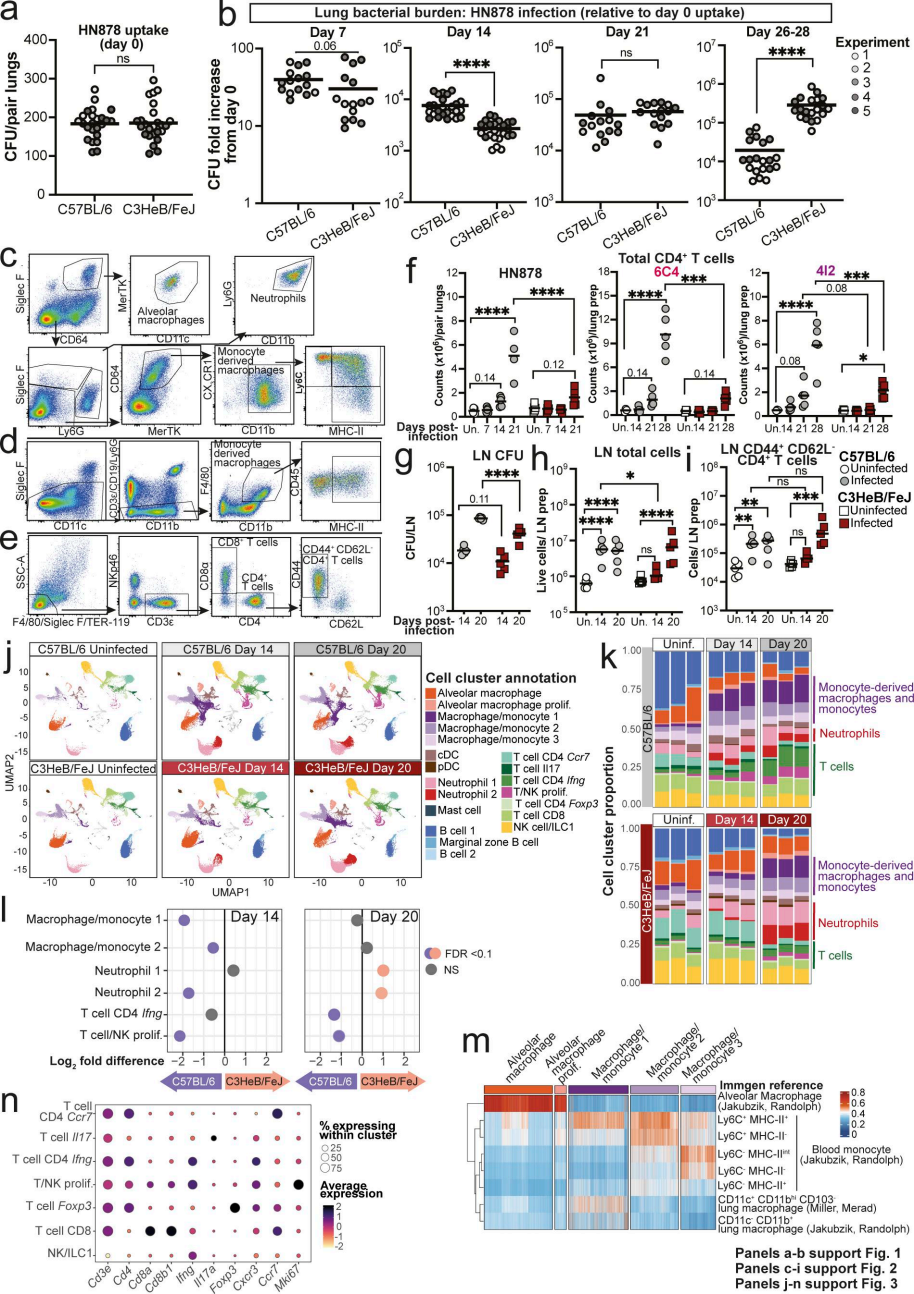
**Analysis of lung and lymph node early during *M. tuberculosis* infection.** C57BL/6 and C3HeB/FeJ mice were aerosol infected with *M. tuberculosis* HN878, 6C4, or 4I2, and infection and immune parameters were assessed. **(a)** Lung CFU counts taken immediately after infection of C57BL/6 and C3HeB/FeJ mice with HN878 in five independent experiments. **(b)** Lung CFU counts at different time points after HN878 infection, represented as fold increases from the mean CFU in the respective age-, sex-, and strain-matched mice analyzed immediately after infection. Panels a and b show data from three to five independent experiments with *N* = 5 mice per group are overlaid per time point with lines at the grand mean. Statistical analysis in a and b shows mouse strain effects across pooled experiments as determined by two-way ANOVA. **(c–e)** Representative flow cytometry dot plots showing the gating strategies used to identify: (c) myeloid cell populations; (d) MDMs specifically in 6C4 and 4I2 experiments; (e) T cell populations. **(f)** Numbers of total CD4^+^ T cells (CD3ε^+^CD45^+^) in lung tissue as determined by flow cytometry. Points show individual mice with lines at the mean. Data are from single experiments with three to five mice per group that are representative of two independent experiments. **(g)** CFU counts in lung-draining lymph node (LN) tissue. Data are from a single experiment with five mice per group, showing individual points with lines at the mean. Data are representative of two independent experiments. Statistical analysis in f and g: two-way ANOVA with Holm–Sidak post hoc test. **(h)** Total live cells in lung-draining lymph nodes. **(i)** Numbers of CD44^+^CD62L^−^ CD4^+^ T cells (CD3ε^+^CD45^+^) in lung-draining lymph nodes as determined by flow cytometry. Panels h and i show points from *N* = 5 mice per group from a single experiment with lines at the median. Data are representative of three independent experiments. Statistical analysis in h and i: aligned ranks transformation two-way ANOVA analysis, with post hoc comparisons between groups shown following Holm’s correction. **(j)** UMAP (Uniform Manifold Approximation and Projection) of integrated and clustered lung leukocyte scRNA-seq data as shown in Fig. 3 a, broken down into individual experimental groups (*N* = 3 per group); cDC, conventional dendritic cell; pDC, plasmacytoid dendritic cell. **(k)** Stacked bar plots showing the relative abundance of each scRNA-seq cluster in each mouse, grouped by broad cell types, with major groups of clusters highlighted. **(l)** Differences in relative abundance of key scRNA-seq clusters between C57BL/6 and C3HeB/FeJ mice at the two infection time points. Differential abundance with false discovery rate <0.1 was taken as statistically significant. **(m)** Reference-based analysis of macrophage and monocyte scRNA-seq clusters against references from the ImmGen database for lung macrophages and blood monocytes. ImmGen contributing investigators for each reference are listed by surname. **(n)** Dot plots showing relative expression of selected marker genes in T and NK cell scRNA-seq clusters. Circle sizes represent the abundance of cells expressing the marker gene, as a percentage of all cells in the cluster within all samples in the analysis. Circle color is proportional to the mean expression of the gene within all cells in the cluster. Panels j–n show data from a single scRNA-seq experiment, and plots show combined data from cells from *N* = 3 mice per group, except for panel k, which shows data from individual mice. Actual or adjusted P values are shown or: *, P < 0.05; **, P < 0.01; ***, P < 0.001; ****, P < 0.0001; ns, not significant.

The primary aim of this study was to identify early differences in the immune response to *M. tuberculosis* infection, preceding distinct disease outcomes and immune responses previously observed during established disease (Moreira-Teixeira et al., 2020b). To this end, relatively TB-resistant C57BL/6 and highly susceptible C3HeB/FeJ mice were analyzed over the first weeks of infection with the highly virulent lineage 2 W/Beijing strain of *M. tuberculosis* HN878, based on our previous report that the blood signature of active TB is recapitulated in C3HeB/FeJ, but not C57BL/6, mice infected with this *M. tuberculosis* strain (Moreira-Teixeira et al., 2020b). C57BL/6 mice displayed an initial spike in lung bacterial burden by 21 days after infection, followed by partial control, while, as expected, TB-susceptible C3HeB/FeJ mice failed to control HN878 infection by the later day 26 time point (Moreira-Teixeira et al., 2020a) (Fig. 1 a; and Fig. S1, a and b). Unexpectedly, higher lung bacterial loads were observed in C57BL/6 as compared with C3HeB/FeJ mice at 14 days after infection (Fig. 1 b), which were not accounted for by differential *M. tuberculosis* uptake during aerosol infection (Fig. S1, a and b), instead suggestive of differences in the very early response to infection between these mouse strains. To verify that this result was not specific to HN878 infection, we additionally infected C57BL/6 and C3HeB/FeJ mice with two lineage 4 isolates, 6C4 and 4I2, known to cause severe and mild TB, respectively, in humans (Sousa et al., 2020). Lower lung bacterial burdens were detected in C3HeB/FeJ than C57BL/6 mice at 14 days after infection with both 6C4 and 4I2 (Fig. 1 b). The mild 4I2 isolate showed the most modest difference in day 14 CFU between mouse strains, suggesting that the magnitude of early differences in lung bacterial burden between C57BL/6 and C3HeB/FeJ mice may be influenced to some extent by the *M. tuberculosis* strain.

To broadly examine the early pulmonary immune response to *M. tuberculosis* infection in C57BL/6 and C3HeB/FeJ mice, bulk RNA-seq was performed on whole lung tissue and total bronchoalveolar lavage (BAL) cells at 14 and 21 days after infection with HN878 and compared with respective uninfected controls. Globally, both lung and BAL analyses revealed a much more pronounced early transcriptional response of immune activation in C57BL/6 than C3HeB/FeJ mice (Fig. 1, c–f), with only minor changes in gene expression compared with uninfected controls in C3HeB/FeJ mice at 14 days after infection. Using k-means clustering to reveal patterns of differential gene expression changes over time in infected mice, we observed that clusters of enriched genes related to macrophage activation, pro-inflammatory cytokine signaling, and antigen presentation were increased from day 14 in C57BL/6 mice, but not until the later day 21 time point in C3HeB/FeJ mice (Fig. 1 d, clusters 7 and 5). Expression of genes associated with effector T cells, including *Ifng*, *Cd4* or *Cd3g* and *Cxcr3*, as well as some type I IFN-stimulated genes (ISGs), was increased earlier and to a greater degree in C57BL/6 than in C3HeB/FeJ mice (Fig. 1, d and f). Conversely, gene clusters showing the highest expression in C3HeB/FeJ mice were only substantially increased by 21 days after infection and were dominated by neutrophil and inflammatory myeloid-associated genes such as *Ly6g*, *Cxcr2*, *S100a8*, and *S100a9* (Fig. 1 d, cluster 6; Fig. 1 f, cluster 5). Together, our initial analysis indicated that C57BL/6 mount a more rapid pulmonary immune response to *M. tuberculosis* infection than C3HeB/FeJ, with a greater contribution from effector T cells and activated macrophages.

### Neutrophils are recruited to lungs of both C57BL/6 and C3HeB/FeJ mice following *M. tuberculosis* infection, while MDM and CD4^+^ T cell accumulation is delayed in C3HeB/FeJ mice

**Figure 2.**
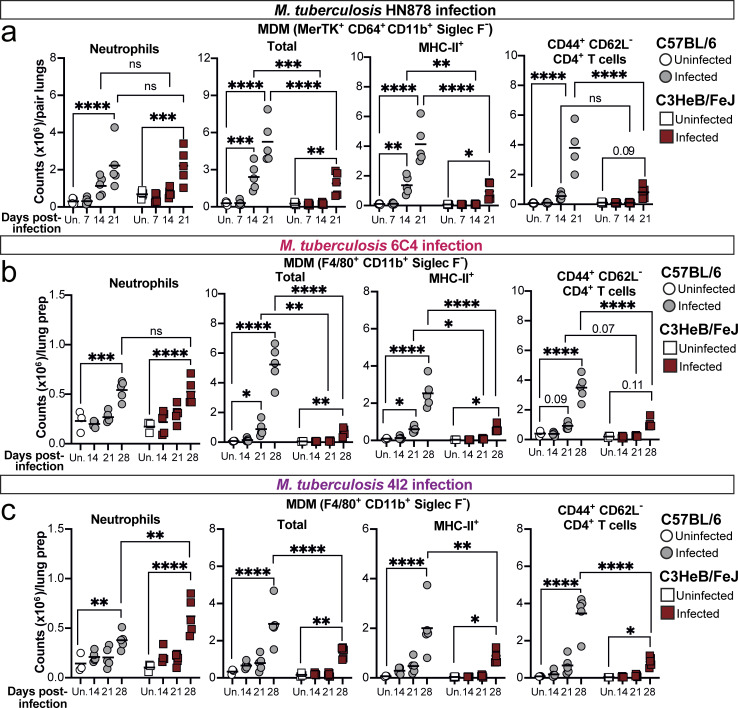
**Neutrophils are recruited to lungs of both C57BL/6 and C3HeB/FeJ mice, while macrophage and CD4** ^**+**^ **T cell accumulation is delayed in TB-susceptible C3HeB/FeJ mice. (a–c)** C57BL/6 and C3HeB/FeJ mice were aerosol infected with (a) HN878, (b) 6C4, or (c) 4I2 *M. tuberculosis* strains, and numbers of neutrophils (Ly6G^hi^CD11b^hi^CD45^+^), total and MHC-II^+^ MDMs (HN878: Siglec F^−^ Ly6G^−^ CD11b^+^CD64^+^MerTK^+^CD45^+^; 6C4/4I2: Siglec F^−^ Ly6G^−^ CD11b^+^F4/80^+^CD45^+^), and CD44^+^CD62L^−^ CD4^+^ T cells (CD3ε^+^CD45^+^) in lung tissue were determined by flow cytometry. Flow cytometry gating was performed as represented in Fig. S1, c–e. Points show individual replicate mice with lines at the mean. Statistical testing: two-way ANOVA with Holm–Sidak post hoc test; actual adjusted P value are shown or: *, P < 0.05; **, P < 0.01; ***, P < 0.001; ****, P < 0.0001; ns, not significant. Data shown are from a single experiment per *M. tuberculosis* strain with *N* = 3–5 mice per group and are representative of a minimum of two independent experiments. See also Fig. S1, c–i.

To verify our bulk RNA-seq findings at the cellular level, we performed flow cytometry analysis of major leukocyte populations in lungs from C57BL/6 and C3HeB/FeJ mice at early time points following HN878 infection (Fig. S1, c–e). Total lung neutrophil numbers were increased to a comparable degree by 21 days after infection in both mouse strains early during HN878 infection (Fig. 2 a, left), which was unexpected in light of the established pathogenic role of neutrophils in TB-susceptible mice, including C3HeB/FeJ, at the peak of disease (Keller et al., 2006; Kimmey et al., 2015; Moreira-Teixeira et al., 2020a; Nandi and Behar, 2011). In contrast, C57BL/6 mice displayed greater early accumulation of Siglec F^−^ CD11b^+^ MDMs than C3HeB/FeJ mice, which was particularly pronounced for the MHC-II^+^ subset (Fig. 2 a, central two panels; Fig. S1 c), likely representing more mature and/or activated cells. It is likely that the MHC-II^–^ Ly6C^+^ subset of MDMs includes differentiating recruited monocytes that have upregulated macrophage markers. This was accompanied by far greater early increases in total (Fig. S1 f) and CD44^+^ CD62L^−^ CD4^+^ T cells (Fig. 2 a, right panel) in C57BL/6 mice. The limited early pulmonary CD4^+^ T cell response in C3HeB/FeJ mice was not due to an absence of live *M. tuberculosis* in lung-draining lymph nodes for T cell priming, since viable *M. tuberculosis* was detectable in both C57BL/6 and C3HeB/FeJ mice at 14 days after infection with HN878 (Fig. S1 g). However, an earlier immune response in lung-draining lymph nodes—as measured by total cellularity and CD44^+^ CD62L^−^ CD4^+^ T cell numbers— was apparent at 14 days after infection in C57BL/6 than in C3HeB/FeJ mice, with similar responses detectable in both mouse strains by day 20 (Fig. S1, h and i).

Comparable results were observed in confirmatory infection experiments with the lineage 4 clinical isolates 6C4 and 4I2 (Fig. 2, b and c). Both C57BL/6 and C3HeB/FeJ mice accumulated lung neutrophils by 28 days after infection, with greater neutrophil numbers in C3HeB/FeJ than C57BL/6 mice following 4I2 infection (Fig. 2, b and c, left panels). The greater early accumulation of total and MHC-II^+^ MDMs in C57BL/6 mice was recapitulated in these models (although identified using a distinct gating strategy from HN878, Fig. S1 d) from days 21 and 28 for the 6C4 and 4I2 infections, respectively (Fig. 2, b and c, central two panels). This was accompanied by markedly increased total (Fig. S1 f) and CD44^+^ CD62L^−^ CD4^+^ T cell (Fig. 2, b and c, right panels) numbers in lungs of C57BL/6 than C3HeB/FeJ mice by 28 days after infection with 6C4 or 4I2.

Thus, independently of the infecting *M. tuberculosis* strain, both C57BL/6 and C3HeB/FeJ mice recruit neutrophils to the lung in the first weeks after infection. However, the eventual superior control of *M. tuberculosis* infection in C57BL/6 mice is preceded by earlier accumulation of lung MDMs and a more rapid and pronounced CD4^+^ T cell response than in C3HeB/FeJ mice.

### scRNA-seq reveals clusters of MDMs and IFN-γ–expressing effector CD4^+^ T cells that accumulate early following *M. tuberculosis* infection in C57BL/6 mice

**Figure 3.**
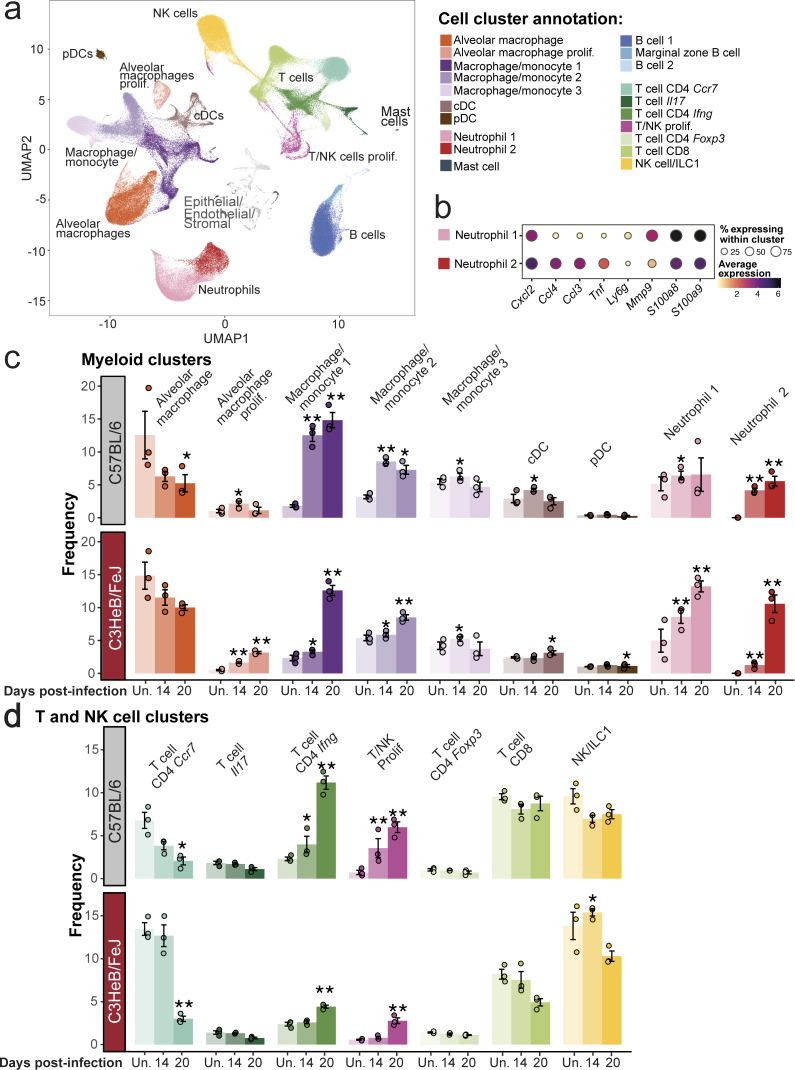
**scRNA-seq reveals earlier increases in monocyte-derived cell and effector CD4** ^**+**^ **T cell subsets in relatively TB-resistant C57BL/6 mice.** C57BL/6 and C3HeB/FeJ mice were aerosol infected with *M. tuberculosis* HN878 and lung CD45^+^ leukocytes enriched at 14 and 20 days after infection, as well as from uninfected controls, fixed, cryo-preserved, and subsequently pooled for scRNA-seq. **(a)** UMAP of integrated and clustered data from all experimental groups (*N* = 3 per group); cDC, conventional dendritic cell; pDC, plasmacytoid dendritic cell. **(b)** Dot plots showing relative expression of selected marker genes in two neutrophil scRNA-seq clusters. Circle sizes represent the abundance of cells expressing the marker gene, as a percentage of all cells in the cluster within all samples in the analysis. Circle color is proportional to the mean expression of the gene within all cells in the cluster. **(c and d)** Differential abundance analysis of (c) myeloid cell clusters and (d) T and NK cell clusters in the different conditions in C57BL/6 and C3HeB/FeJ mice, compared with their respective uninfected controls, as determined by Dirichlet-multinomial regression. *, P < 0.05; **, P < 0.001. Points show individual replicate mice with bars at the mean ± standard error. Data shown are from a single scRNA-seq experiment with *N* = 3 mice per group. See also Fig. S1, j–n.

We next interrogated the precise cellular sources of the distinct early transcriptional responses observed in C57BL/6 and C3HeB/FeJ mice by performing scRNA-seq on enriched CD45^+^ lung leukocytes over the key early window of 14–20 days after infection, as compared with uninfected controls. A total of 197,530 cells was obtained from all samples after filtering, from which a total of 20 leukocyte clusters were derived (Fig. 3 a and Fig. S1 j) and annotated using the clustifyr package, with additional manual annotation guided by published literature.

Two clusters of alveolar macrophages were annotated, the smaller of which had a signature of proliferation and increased modestly in relative abundance with infection in both C57BL/6 and C3HeB/FeJ (Fig. 3, a and c; and Fig. S1, j and k). The nonproliferating alveolar macrophage cluster decreased in relative abundance during infection, reflecting the influx of recruited leukocytes, with a significant decrease in these cells in C57BL/6 mice by day 20 (Fig. 3 c; and Fig. S1, j and k). Three further clusters with monocyte and macrophage transcriptional profiles were identified, likely comprising both monocytes and MDMs (Fig. 3, a and c; and Fig. S1, j and k). Consistent with flow cytometry data, the relative abundance of these “macrophage/monocyte” clusters was more markedly increased at 14 days after infection in C57BL/6 as compared with C3HeB/FeJ mice, with scRNA-seq further revealing the macrophage/monocyte 1 and, to a lesser extent, macrophage/monocyte 2 cluster, to underpin the earlier increase in C57BL/6 mice (Fig. 3 c and Fig. S1, j–l). Comparison of our scRNA-seq clusters to ImmGen lung macrophage and blood monocyte signatures (Gautier et al., 2012) indicated that the macrophage/monocyte 1 cluster had the greatest similarity to steady-state lung CD11b^+^ macrophages, as well as similarity to Ly6C^+^MHC-II^+^ blood monocytes (Fig. S1 m), consistent with these representing the most mature and/or activated MDMs in our dataset. The macrophage/monocyte 2 and 3 clusters showed the greatest similarity to Ly6C^+^ and Ly6C^−^ blood monocyte transcriptomes, respectively (Fig. S1 m), suggesting that these clusters represent monocytes, which may have migrated into the lung, and/or less mature MDMs.

Two distinct neutrophil clusters were identified, which differed in their expression of several marker genes, suggestive of different populations or functional states (Fig. 3, a and b). Both neutrophil clusters increased in abundance as infection progressed in both strains of mice (Fig. 3 c; and Fig. S1, j and k), recapitulating flow cytometry results (Fig. 2 a). The neutrophil 1 cluster was similarly abundant in C57BL/6 and C3HeB/FeJ mice at 14 days after infection but then increased further in C3HeB/FeJ mice by day 20 (Fig. 3 c and Fig. S1, j–l). In contrast, the neutrophil 2 cluster was more abundant in C57BL/6 than C3HeB/FeJ mice at day 14 but then strikingly increased to represent a greater proportion of lung leukocytes in C3HeB/FeJ mice by day 20 (Fig. 3 c and Fig. S1, j–l), suggesting that this cluster is associated with progression toward more severe TB disease. Accordingly, neutrophil 2 expressed higher levels of cytokine and chemokine genes, such as *Cxcl2*, *Ccl3*, *Ccl4*, and *Tnf*, but slightly lower expression of hallmark neutrophil marker genes *Mmp9*, *Ly6g*, *S100a8*, and *S100a9* than neutrophil 1 (Fig. 3 b), consistent with a more pro-inflammatory and potentially pathogenic neutrophil population.

Seven major T and natural killer (NK) cell clusters were identified (Fig. 3 a and Fig. S1 j), including a population resembling naïve, circulating CD4^+^ T cells (T cell CD4 *Ccr7*; Fig. S1 n) that decreased in frequency as infection progressed in both mouse strains, albeit earlier in C57BL/6 mice (Fig. 3 d; and Fig. S1, j and k). In contrast, the T cell CD4 *Ifng* and T/NK Prolif clusters increased in frequency earlier and to a greater degree during infection in C57BL/6 than C3HeB/FeJ mice (Fig. 3 d and Fig. S1, j–l). We observed these populations to have the highest *Ifng* expression among T cell clusters, consistent with effector CD4 T cell identity (Fig. S1 n), although the T/NK Prolif cluster, which had a clear signature of proliferation (Fig. S1 n), also encompassed a small proportion of cells clustering with NK cells (Fig. 3 a). No clear differences in the changes in relative abundance during infection were observed between mouse strains for the small *Il17a*-expressing T cell *Il17* cluster; the T cell CD4 *Foxp3* cluster, likely reflecting regulatory T cells; or the CD8 T cell and NK/ILC1 clusters (Fig. 3 d; and Fig. S1, j, k, and n).

Thus, our initial scRNA-seq analysis showed that the enhanced early immune response to *M. tuberculosis* in relatively TB-resistant C57BL/6 mice is distinguished by accumulation of mature/activated MDMs and IFN-γ–producing effector T cells, with limited progression of the pro-inflammatory neutrophil response, while neutrophil activation is exacerbated as infection progresses in C3HeB/FeJ mice.

### Chemokine signals favoring neutrophil, rather than T cell, recruitment dominate lungs of TB-susceptible mice early in infection

**Figure 4.**
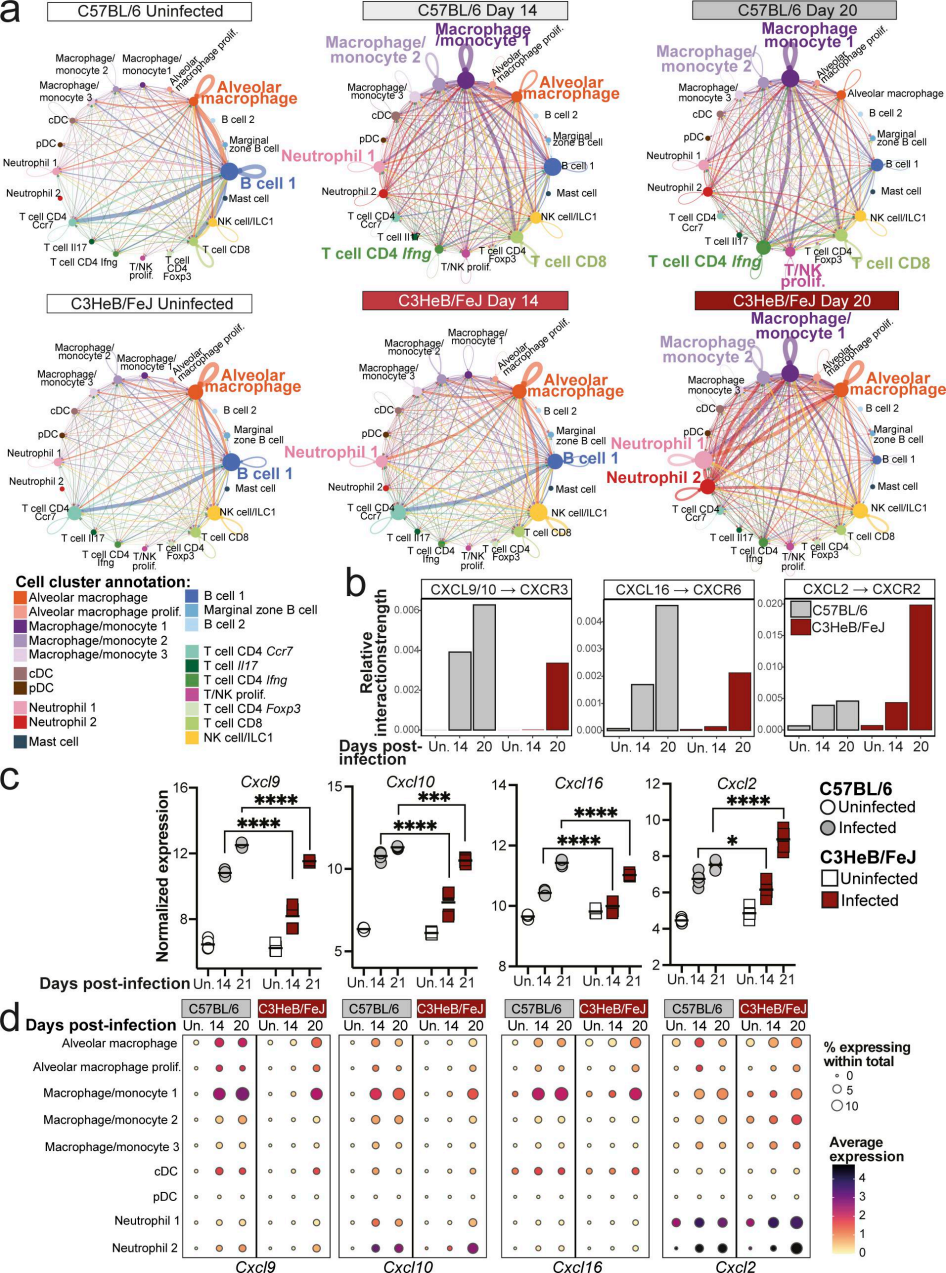
**Myeloid–T cell chemokine interactions dominate lungs of C57BL/6 mice, while neutrophil recruitment is favored in TB-susceptible C3HeB/FeJ mice early during infection.** Leukocyte clusters from our scRNA-seq dataset were subjected to CellChat analysis to infer cell–cell interactions, and expression of chemokine genes was examined in bulk and scRNA-seq data. **(a)** Circle plots showing predicted interaction strength between cell populations in the different conditions. Line colors indicate the inferred signal-sending population, line thickness is proportional to communication probability, and circle size is proportional to cell type/cluster abundance. **(b)** Bar plots showing the relative contribution of the indicated receptor/ligand pairs to total inferred interaction activity in each group. **(c)** DESeq2-normalized expression values of the indicated chemokine genes from bulk RNA-seq analysis of whole lung tissue. Points show individual replicate mice with lines at the mean. Statistical analysis: two-way ANOVA with Holm–Sidak post hoc test; *, P < 0.05; ***, P < 0.001; ****, P < 0.0001. **(d)** Dot plots showing expression of the indicated genes in myeloid cell populations in scRNA-seq data. Circle sizes represent the abundance of cells expressing the gene, as a percentage of total cells. Circle color is proportional to the mean expression of the gene within all cells in the cluster. Data in panels a, b, and d are from a single scRNA-seq experiment, with plots showing combined data from cells from *N* = 3 mice per group. Data in panel c are from a single bulk RNA-seq experiment with *N* = 5 mice per group. See also Data S1 and Fig. S2.

We next leveraged our scRNA-seq dataset to predict cell-to-cell interactions occurring in resistant and susceptible mice at early time points after infection, using the R package CellChat, which infers active cell-to-cell interactions based on ligand and receptor gene expression (Jin et al., 2021). CellChat predicted alveolar macrophages and B cells as the most likely sources of cell–cell interaction signals in naïve lungs from both C57BL/6 and C3HeB/FeJ mice (Fig. 4 a), reflecting the relative abundance of these cell populations in lungs prior to infection (Fig. 3 c; and Fig. S1, j and k). By 14 days after infection in C57BL/6 mice, substantial interaction activity was predicted from the macrophage/monocyte 1 and 2 clusters, signaling toward neutrophil and T cell populations (Fig. 4 a). The strength of these inferred macrophage–T cell interactions was further increased in C57BL/6 mice by 20 days after infection, while inferred macrophage–neutrophil interactions remained comparable with day 14 (Fig. 4 a). Inferred cell–cell interactions in C3HeB/FeJ mice at 14 days after infection were similar to those in uninfected controls, consistent with their limited early pulmonary immune response, although inferred signaling from alveolar macrophages to the neutrophil 1 and macrophage/monocyte 2 and 3 clusters of monocyte-like cells was modestly increased, suggestive of an early innate response to infection (Fig. 4 a). Inferred interaction strength in C3HeB/FeJ mice increased substantially by 20 days after infection but was dominated by predicted signaling of macrophage/monocyte 1 and 2 and alveolar macrophage clusters to neutrophils, including the pro-inflammatory neutrophil 2 cluster, as well as interactions within these cell clusters, with relatively little signaling to T cells predicted compared with C57BL/6 mice (Fig. 4 a).

**Figure S2.**
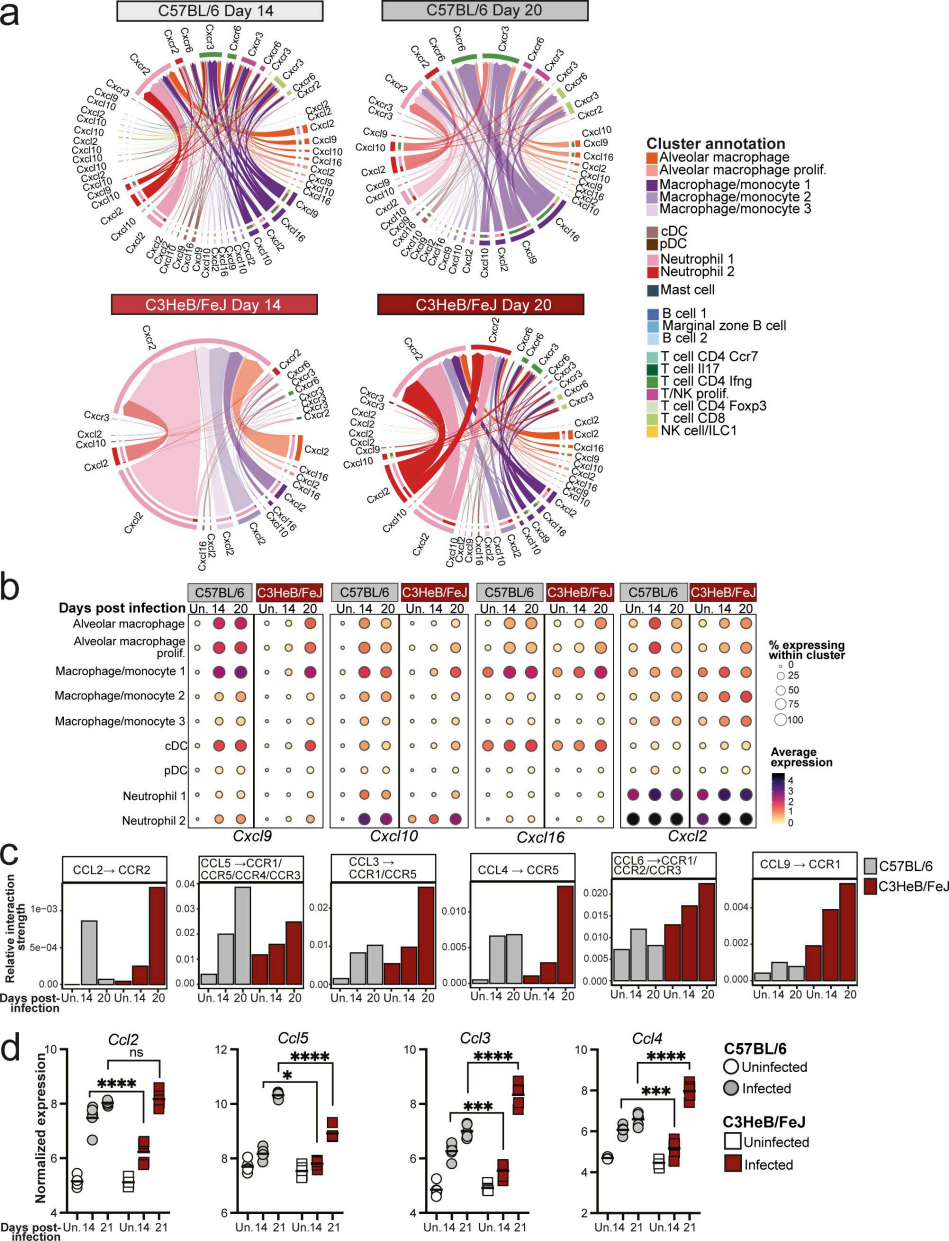
**Distinct early chemokine expression in lungs of C57BL/6 and C3HeB/FeJ mice. (a)** Chord plots showing inferred interaction strength via the indicated receptor–ligand pairs between cell populations in the different conditions. Outer circle and arrow colors indicate the predicted sending population, and the inner circle colors indicate the predicted receiving populations. Arrow thickness indicates the overall signaling contribution. **(b)** Dot plots showing expression of the indicated genes in myeloid cell populations in scRNA-seq data. Circle sizes represent the abundance of cells expressing the gene, as a percentage of cells within the cluster. Circle color is proportional to the mean expression of the gene within all cells in the cluster. **(c)** Bar plots showing the relative contribution of the indicated receptor/ligand interactions to total inferred interaction activity in each group. Data in a–c are from a single scRNA-seq experiment, and plots show combined data from cells from *N* = 3 mice per group. **(d)** DESeq2-normalized expression values of the indicated chemokine genes in whole lung. Data shown are from individual mice from a single bulk RNA-seq experiment with *N* = 5 mice per group and lines at the mean. Statistical analysis: two-way ANOVA with Holm–Sidak post hoc test: *, P < 0.05; ***, P < 0.001; ****, P < 0.0001; ns, not significant.

We further interrogated our CellChat results by pathway analysis, identifying pathways with predicted differential activity in C57BL/6 and C3HeB/FeJ mice at each time point. Several pathways identified reflected differential expression of ligand genes in macrophage populations between the mouse strains across all time points, suggestive of genetically determined diversity (Data S1 a). These included Apolipoprotein E, with broadly higher *Apoe* expression observed in C57BL/6 mice, whereas expression of ligand genes for the SPP1, sialoadhesin, and annexin pathways was higher in C3HeB/FeJ mice, including within alveolar macrophages (Data S1, a–d). Focusing on pathways with predicted differential activity during infection, the CXCL chemokine CellChat pathway was of particular interest, with higher predicted activity in C57BL/6 mice at day 14 and, subsequently, higher activity in C3HeB/FeJ mice at day 20 (Data S1 a), which was driven predominantly by four receptor/ligand pairs (Fig. S2 a). Signaling of CXCL9 and CXCL10 via CXCR3, a well-established axis in T cell recruitment, particularly Th1 cells (Karin, 2020), was predicted to be elevated earlier and to a greater degree in C57BL/6 than C3HeB/FeJ mice (Fig. 4 b and Fig. S2 a). Similar results were inferred for the CXCL16-CXCR6 axis (Fig. 4 b and Fig. S2 a), which is involved in localization of T cells, particularly T-resident memory cells, within tissues (Mabrouk et al., 2022). Predicted signaling for both pathways was strongest from the macrophage/monocyte 1 MDM cluster to the T cell CD4 IFN-γ cluster (Fig. S2 a), supporting a role in mediating protective MDM–CD4^+^ T cell interactions in relatively TB-resistant mice. Accordingly, expression of *Cxcl9*, *Cxcl10*, and *Cxcl16* was increased earlier, at 14 days after infection in lungs of C57BL/6 compared with C3HeB/FeJ mice (Fig. 4 c), with expression most pronounced in macrophages, conventional dendritic cells (cDCs), and, in the case of *Cxcl10*, neutrophils (Fig. 4 d and Fig. S2 b). CellChat-predicted pathway activity is in part influenced by abundance of sending and receiving cell populations. However, we also found the average expression of *Cxcl9* and *Cxcl10* to be higher in macrophage clusters from C57BL/6 compared with C3HeB/FeJ mice (Fig. S2 b), suggesting that both MDM abundance and the relative expression of these chemokines by MDMs contribute to greater potential for MDM–T cell interactions in the resistant mice.

While early activity of the key neutrophil-attractant CXCL2-CXCR2 axis was predicted in C57BL/6 mice, predicted activity did not increase further between 14 and 20 days after infection (Fig. 4 b). In contrast, inferred activity of this pathway increased starkly to become dominant in C3HeB/FeJ mice at 20 days after infection (Fig. 4 b), driven by high predicted signaling activity of *Cxcl2* between neutrophils, as well as from the macrophage/monocyte 1 and 2 clusters to neutrophils (Fig. S2 a). Expression of *Cxcl2* in whole lung tissue mirrored this trend, and scRNA-seq confirmed the highest *Cxcl2* expression to be in the inflammatory neutrophil 2 cluster, although high expression was observed across all monocyte, macrophage, and neutrophil clusters (Fig. 4, c and d; and Fig. S2 b).

Increased early predicted chemokine signaling activity in C57BL/6 mice was also predicted for the CCL pathway (Data S1 a). Specifically, an earlier increase in the monocyte-attractant CCL2–CCR2 axis was predicted in C57BL/6 than C3HeB/FeJ mice (Fig. S2 c), consistent with their earlier accumulation of MDMs during infection (Fig. 2 a and Fig. 3 c). Greater CCL5 activity was predicted in C57BL/6 mice at 20 days after infection, whereas predicted activity of the chemokines CCL6, CCL9, CCL3, and CCL4 was highest in C3HeB/FeJ mice by 20 days after infection (Fig. S2 c). Accordingly, we observed *Ccl3* and *Ccl4* to be highly expressed in the pro-inflammatory neutrophil 2 cluster (Fig. 3 b) that is highly abundant in C3HeB/FeJ mice at this time point (Fig. 3 c and Fig. S1, j–l). Kinetics of expression of CCL chemokines in whole lung tissue was consistent with these differential findings in C57BL/6 and C3HeB/FeJ mice (Fig. S2 d).

Collectively, using bulk and scRNA-seq data, we have shown dynamic and distinct lung chemokine expression in C57BL/6 and C3HeB/FeJ mice in the first weeks of infection. While C57BL/6 mice expressed *Cxcl9*, *Cxcl10*, and *Cxcl16*, favoring T cell recruitment, C3HeB/FeJ mice expressed late and higher levels of pro-inflammatory chemokines *Cxcl2*, *Ccl3*, and *Ccl4*, corresponding to their sharp increase in inflammatory neutrophils.

### Neutrophils are spatially segregated from CD4^+^ T cells in TB lesions and dominate early lesions in TB-susceptible mice

**Figure 5.**
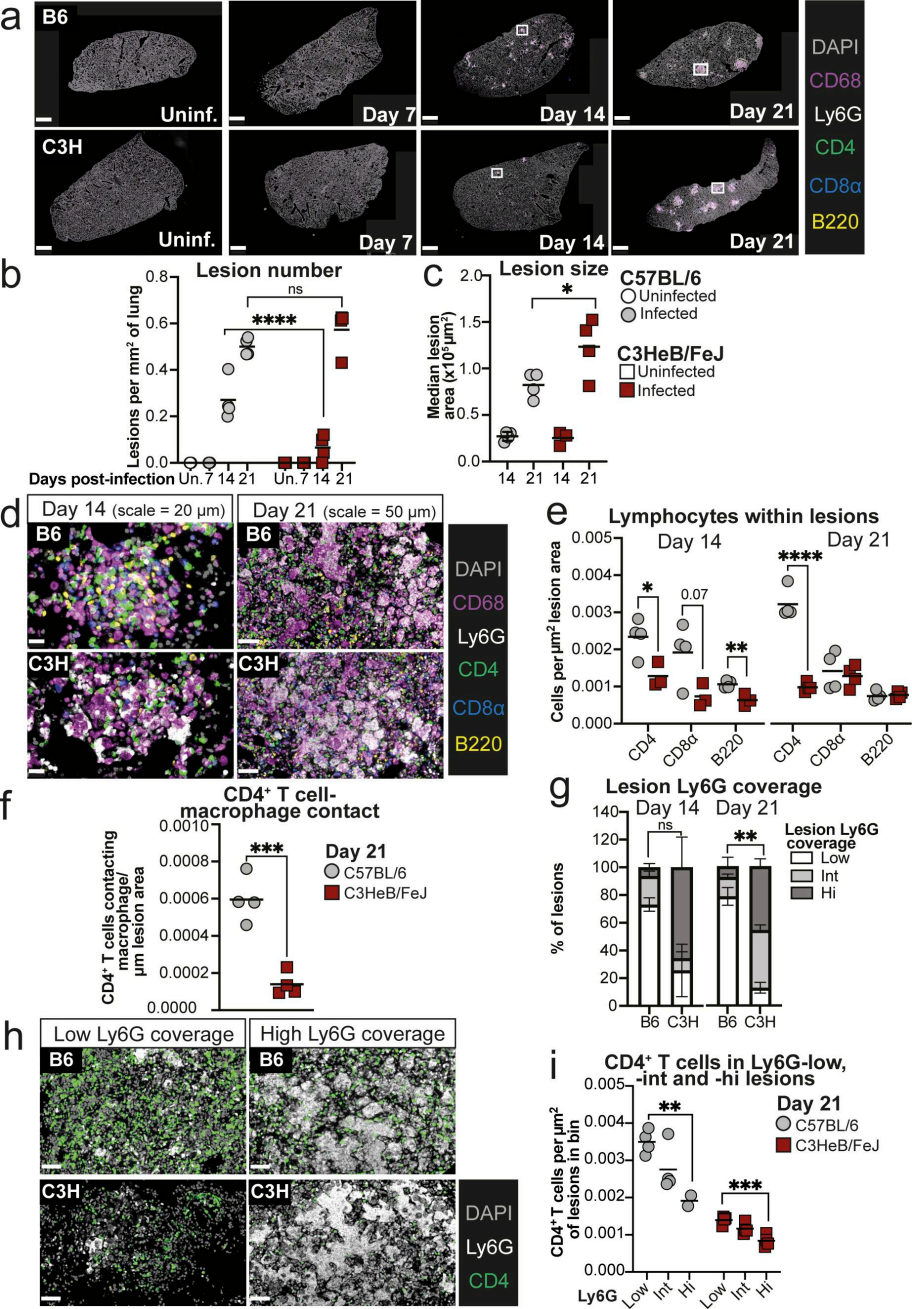
**Spatial separation of CD4** ^**+**^ **T cells and neutrophils in TB lesions.** C57BL/6 (B6) and C3HeB/FeJ (C3H) mice were aerosol infected with *M. tuberculosis* HN878 and lungs harvested for multiparameter immunofluorescence staining of formaldehyde-fixed, paraffin-embedded sections at the indicated time points. **(a)** Representative low-power images of lung lobes, showing distribution of lesions. White boxes indicate areas at days 14 and 21 shown at greater magnification in panel d. The images from days 14 and 21 are reproduced in Fig. S3 a, along with those from the other replicate mice at these time points. Scale bars = 1 mm. **(b)** Quantification of numbers of lesions in whole lungs, normalized for tissue area. **(c)** Median area of lesions detected per mouse. **(d)** Representative images of lesions at 14 and 21 days after infection, showing all immune cell markers. Images are shown at different scales to aid visualization (day 14 scale = 20 µm; day 21 scale = 50 µm). **(e)** Number of cells positive for the indicated markers within lesions, normalized for the total area of all lesions. **(f)** Numbers of CD4^+^ T cells in contact with a macrophage annotation (≤0 μm distance), normalized for the total area of all lesions. **(g)** Stacked bar plots showing percentages of lesions across whole lungs falling into low (≤20%), intermediate (Int, >20% ≤40%), or high (Hi, >40) bins for coverage with Ly6G staining. Data shown are means ± standard error of all mice with detectable lesions (*N* = 3 for day 14 C3HeB/FeJ; *N* = 4 for others). **(h)** Representative images showing the relative distribution of CD4^+^ T cells and Ly6G staining in lesions with low and high Ly6G coverage. Scale bar = 50 μm. **(i)** Number of CD4^+^ T cells within lesions in the different Ly6G coverage bins at 21 days after infection, normalized for the total area of lesions analyzed per bin. Plots in b, c, e, f, and i show points representing all individual replicate mice with detectable lesions, with lines at the mean. Data shown are from a single experiment with *N* = 4 mice per group and are representative of two independent experiments. Statistical analysis: b, c, and e, two-way ANOVA with Holm–Sidak post hoc test; f, unpaired *t* test; g, Dirichlet-multinomial regression, with the indicated P values corresponding to the mouse strain effect on frequency of Ly6G-high lesions; i, one-way ANOVA with Holm–Sidak post hoc test. Actual adjusted *P* values are shown or: *, P < 0.05; **, P < 0.01; ***, P < 0.001; ****, P < 0.0001. See also: Fig. S3, a–c.

**Figure S3.**
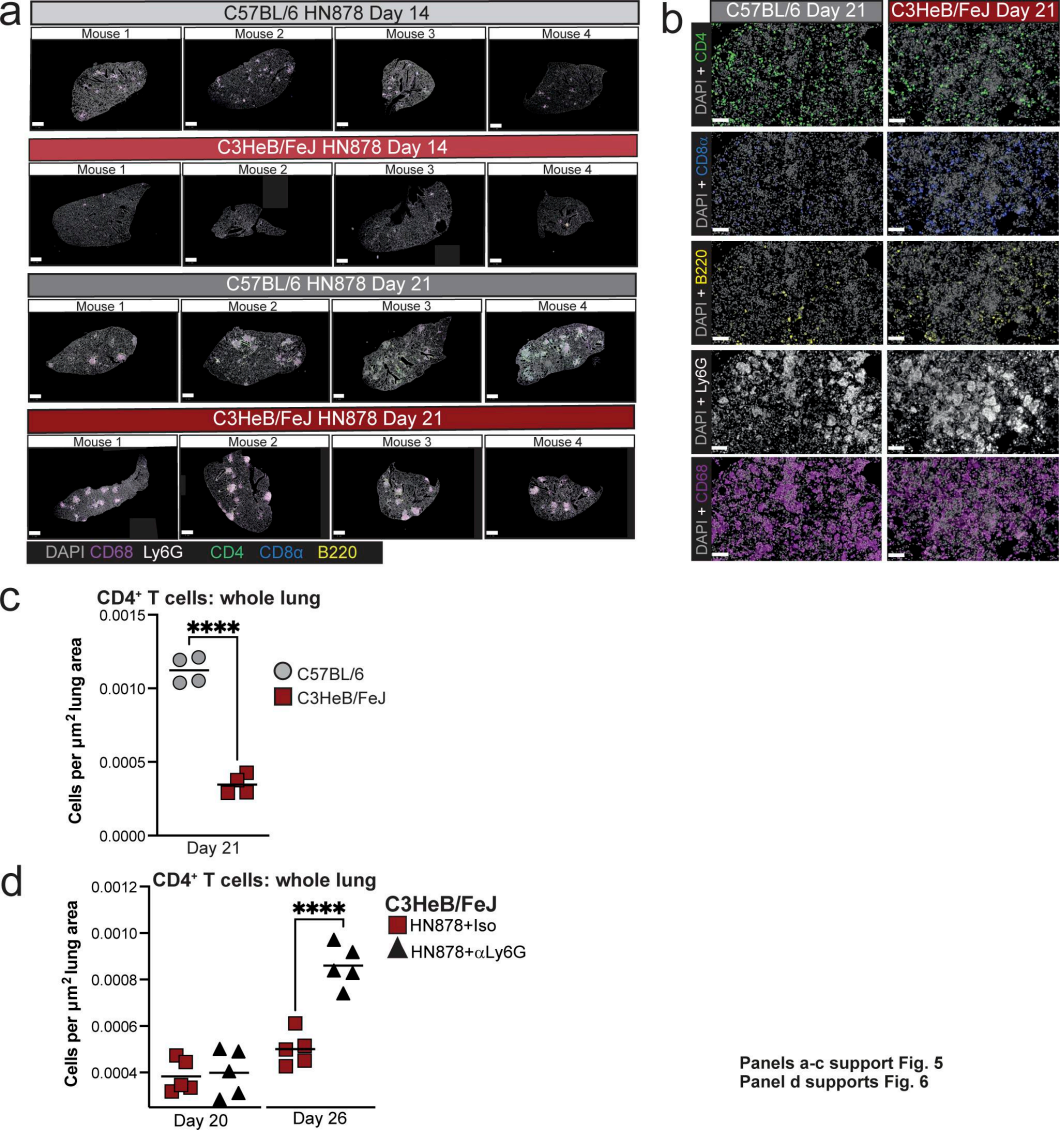
**Distinct early lung lesion composition in C57BL/6 and C3HeB/FeJ mice. (a)** Additional representative lung lobe images of multiplex immunofluorescence analysis from each mouse at early time points after HN878 infection. Images from the mice at 14 and 21 days after infection shown in Fig. 5 a are also reproduced here. Scale bar = 1 mm. **(b)** Images showing individual fluorescent antibody staining corresponding to the images in Fig. 5 d. Scale bar = 50 μm. **(c and d)** CD4^+^ T cell numbers in whole left lung lobes from experiments shown in Figs. 5 and 6, respectively, normalized to tissue area. Data shown are from single experiments with *N* = 4–5 mice per group and are representative of two independent experiments. Plots show individual mice with lines at the mean. Statistical analysis: unpaired *t* test; ****, P < 0.0001.

The observation that MDMs and neutrophils displayed distinct early chemokine expression in *M. tuberculosis*–infected C57BL/6 and C3HeB/FeJ mice prompted us to investigate leukocyte infiltration into the developing lung lesions. Using multiparameter immunofluorescence staining, we first examined the overall distribution of pathology across lung tissue, identifying TB lesions as clusters of CD68^+^ macrophages in lung parenchyma. By 14 days after infection, we observed large numbers of early lesions in C57BL/6 mice, but far fewer lesions in C3HeB/FeJ mice (Fig. 5, a and b; and Fig. S3 a), consistent with the increased MDM numbers observed at this time point in C57BL/6 in whole lungs (Fig. 2 a and Fig. 3 c). Despite their reduced frequency in C3HeB/FeJ mice, the median size of lesions detected at day 14 was similar in both mouse strains (Fig. 5, c and d). By 21 days after infection, comparable lesion numbers were observed in both mouse strains, with generally larger lesions observed in C3HeB/FeJ mice (Fig. 5, a–c and Fig. S3 a).

We next assessed leukocyte composition of early TB lesions, using Ly6G as a marker of neutrophils and CD4, CD8α, and B220 as markers of CD4^+^ T cells, CD8^+^ T cells, and B cells, respectively (Fig. 5 d and Fig. S3 b). Even at the early day 14 time point, we observed greater density of lymphocytes within TB lesions of C57BL/6 than C3HeB/FeJ mice (Fig. 5, d and e). By day 21, there was a much more pronounced increase in CD4^+^ T cells in C57BL/6 mice (Fig. 5, d and e). Greater CD4^+^ T cell abundance was observed across whole lung tissue of infected C57BL/6 mice, as well as in TB lesions (Fig. S3 c). The greater CD4^+^ T cell density in early C57BL/6 lesions resulted in more extensive interactions between CD4^+^ T cells and CD68^+^ macrophages (Fig. 5 f), consistent with increased potential for protective immune cell interactions in the C57BL/6 lesions.

We observed heterogeneity in the degree of Ly6G staining coverage between lesions within individual mice at these early time points. Most C57BL/6 mouse lesions displayed relatively low coverage with Ly6G staining, whereas almost all C3HeB/FeJ lesions had intermediate to high Ly6G coverage, suggestive of large aggregates or swarms of neutrophils, by 21 days after infection (Fig. 5, d and h) during their progression to severe disease. CD4^+^ T cells showed minimal co-localization with areas of extensive Ly6G staining within lesions (Fig. 5, d, h, and i). Accordingly, a clear inverse relationship was observed in lesions from C57BL/6 mice, with higher CD4^+^ T cells numbers observed in Ly6G-low lesions than in the minority with high Ly6G coverage (Fig. 5, h and i). CD4^+^ T cell numbers were lower in all lesion classes from C3HeB/FeJ than from C57BL/6 mice, but higher CD4^+^ T cell density was observed in the minority of smaller lesions with low Ly6G coverage compared with Ly6G-hi lesions (Fig. 5, h and i). Together, our spatial analysis revealed that CD4^+^ T cells are underrepresented in neutrophil-dense lesion areas and that the low effector CD4^+^ T cell numbers observed in lungs of *M. tuberculosis*–infected C3HeB/FeJ mice is reflected in the rapid domination of lesions by neutrophil-dense pathology, with a marked failure to accumulate CD4^+^ T cells in the vicinity of infected cells.

### Neutrophils limit macrophage activation and CD4^+^ T cell accumulation in lungs of TB-susceptible mice

**Figure 6.**
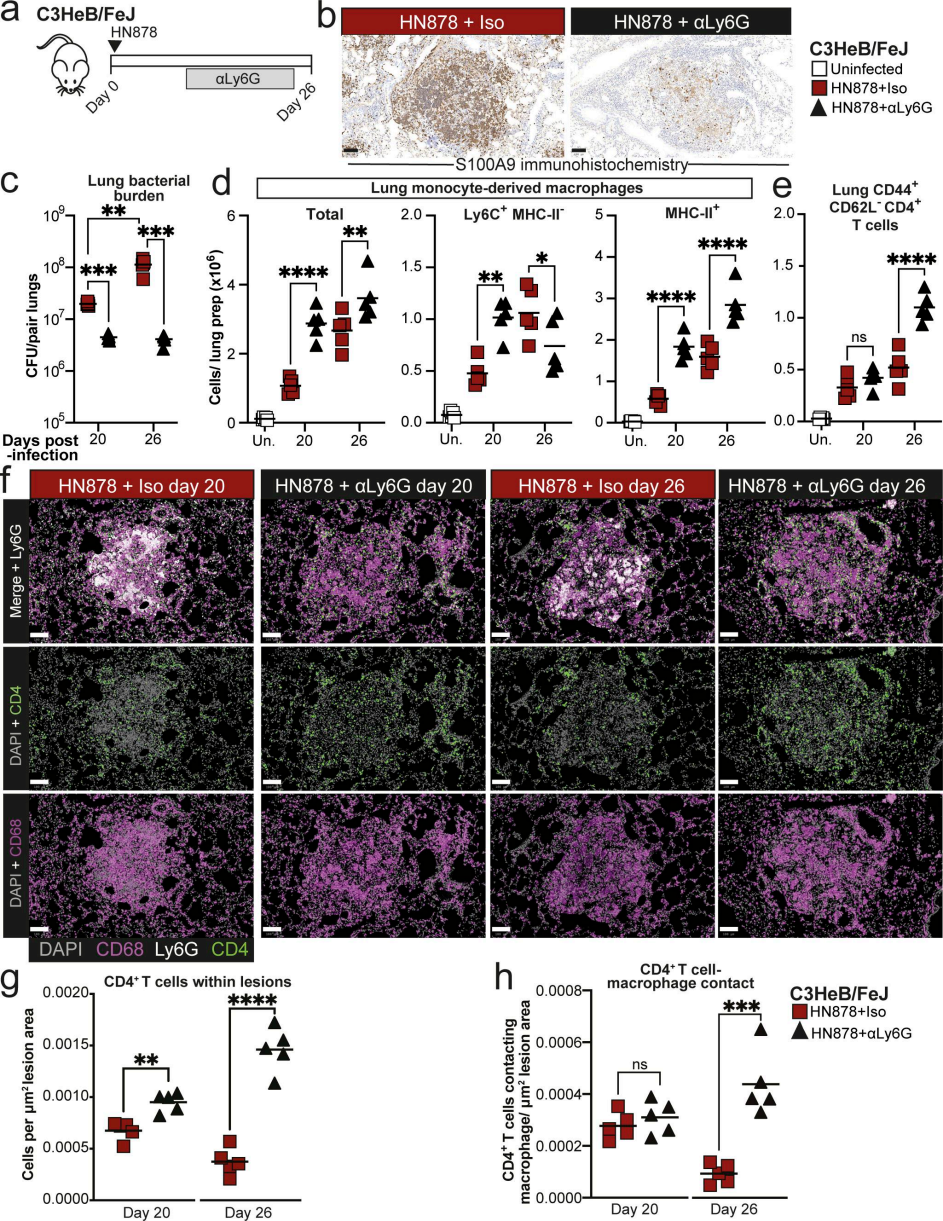
**Neutrophil depletion increases macrophage activation and CD4** ^**+**^ **T cell numbers in lung lesions in TB-susceptible C3HeB/FeJ mice. (a)** C3HeB/FeJ mice were aerosol infected with *M. tuberculosis* HN878 and received intraperitoneal injection of either anti-Ly6G (αLy6G) or isotype control three times per week between days 12 and 25. Tissues were analyzed at 20 and 26 days after infection. **(b)** Representative images of S100A9 immunohistochemistry in lung sections at 20 days after infection, confirming neutrophil depletion in αLy6G-treated mice. Scale bars = 100 μm. **(c)** Lung *M. tuberculosis* CFU counts. **(d)** Numbers of total, Ly6C^+^MHC-II^−^ and MHC-II^+^ MDMs (Siglec F^−^ Ly6G^−^ CD11b^+^CD64^+^MerTK^+^CD45^+^) in lung tissue as determined by flow cytometry. **(e)** Numbers of CD44^+^ CD62^−^ CD4^+^ T cells (CD3ε^+^CD45^+^) in lung tissue as determined by flow cytometry. **(f)** Representative images of lung lesions showing macrophage (CD68, magenta), CD4^+^ T cells (CD4, green), and neutrophil (Ly6G, white) staining at 20 and 26 days after infection. Scale bars = 100 μm. **(g)** Number of CD4^+^ T cells within lung lesions, normalized for the total area of all lesions across whole left lungs. **(h)** Numbers of CD4^+^ T cells in contact with a macrophage annotation (≤0 μm distance), normalized for the total area of all lesions across whole left lungs. Points show individual replicate mice with lines at the mean. Data shown are from a single experiment with *N* = 5 mice per group, representative of two independent experiments. Statistical analysis: (c–e) two-way ANOVA with Holm–Sidak post hoc test; (g and h) unpaired t-test: *, P < 0.05; **, P < 0.01; ***, P < 0.001; ****, P < 0.0001; ns, not significant. See also: Fig. S3 d.

Given the relative paucity of CD4^+^ T cells in lung lesion areas high in neutrophils, we next asked whether the absence of neutrophils would allow greater CD4^+^ T cell infiltration of lesions and activation of macrophages in C3HeB/FeJ mice. To address this, we administered anti-Ly6G or isotype control antibodies from around the point of early lesion formation in C3HeB/FeJ mice, with neutrophil depletion confirmed by marked reduction of S100A9^+^ cells in lungs (Fig. 6, a and b). Consistent with previous data (Moreira-Teixeira et al., 2020a), anti-Ly6G treatment substantially reduced lung bacterial burden at the peak of disease at 26 days after infection, and we additionally observed an earlier reduction at 20 days after infection in the neutrophil-depleted mice (Fig. 6 c). Protection of anti-Ly6G–treated mice was accompanied by an increase in total MDM numbers at 20 and 26 days after infection (Fig. 6 d). MDMs with a less mature, inflammatory monocyte-like, Ly6C^+^ MHC-II^−^ surface phenotype were increased at day 20 but decreased at day 26 in anti-Ly6G–treated compared with control antibody-treated mice, whereas MHC-II^+^ MDMs were more abundant at both time points (Fig. 6 d), suggestive of increased activation of incoming MDMs to an MHC-II^+^ phenotype with time during infection in the context of neutrophil depletion.

Increased MDM activation coincided with significantly increased numbers of CD44^+^CD62L^−^CD4^+^ T cells in lungs of anti-Ly6G–treated mice compared with isotype controls by 26 days after infection (Fig. 6 e) and increased frequency of CD4^+^ T cells within lung lesions as early as 20 days after infection, which was greatly augmented by day 26 (Fig. 6, f and g). Total lung CD4^+^ T cell numbers were also increased in whole lung with neutrophil depletion, but this occurred later than in the lesions, increasing with neutrophil depletion at day 26 but not day 20 (Fig. S3 d). The number of CD4^+^ T cell–macrophage contacts were also increased at day 26 in lesions of anti-Ly6G–treated mice (Fig. 6 h). Thus, in the absence of neutrophils, C3HeB/FeJ mice form more extensive CD4^+^ T cell–macrophage interactions in lesions and show evidence of increased MDM activation.

### C57BL/6 mice display a higher early lung type I IFN response signature than C3HeB/FeJ mice during *M. tuberculosis* infection, accompanying that of protective cytokines

**Figure 7.**
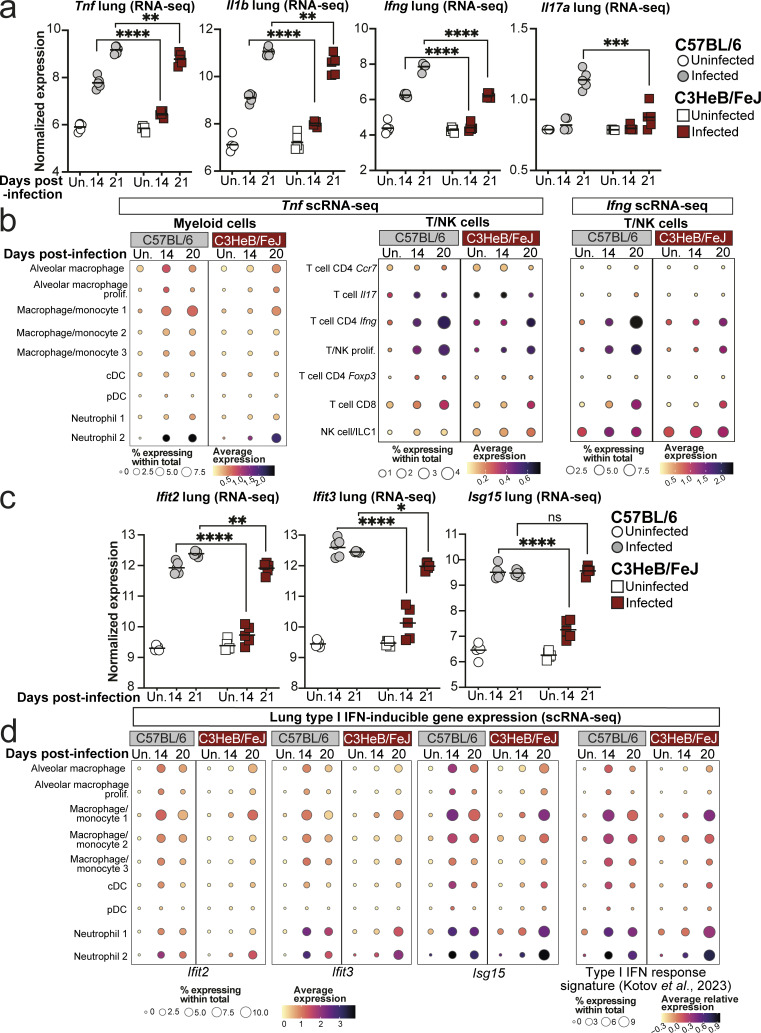
**Early lung type I IFN response signature is higher in C57BL/6 than in susceptible C3HeB/FeJ mice.** Bulk and single cell lung RNA-seq data were interrogated for expression of cytokine and cytokine response genes. **(a)** DESeq2-normalized expression values of the indicated cytokine genes in whole lung bulk RNA-seq data. **(b)** Dot plots showing expression of *Tnf* and *Ifng* in the indicated cell populations in scRNA-seq data. **(c)** DESeq2-normalized expression values of the indicated ISGs in whole lung bulk RNA-seq data. **(d)** Dot plots showing expression of individual representative ISGs or a 37-gene type I IFN response signature (Kotov et al., 2023) in myeloid cell populations in scRNA-seq data. Data shown in panels a and c are from a single bulk RNA-seq experiment with *N* = 5 mice per group. Points represent individual replicate mice as points with lines at the mean. Statistical analysis: two-way ANOVA with Holm–Sidak post hoc test: *, P < 0.05; **, P < 0.01; ***, P < 0.001; ****, P < 0.0001; ns, not significant. Data in panels b and d are from a single scRNA-seq experiment, and plots show combined data from cells from *N* = 3 mice per group. Circle sizes represent the abundance of cells expressing the gene, as a percentage of total cells. Circle color is proportional to the mean expression of the gene or signature within all cells in the cluster. See also Fig S4 and Fig. S5 a.

**Figure S4.**
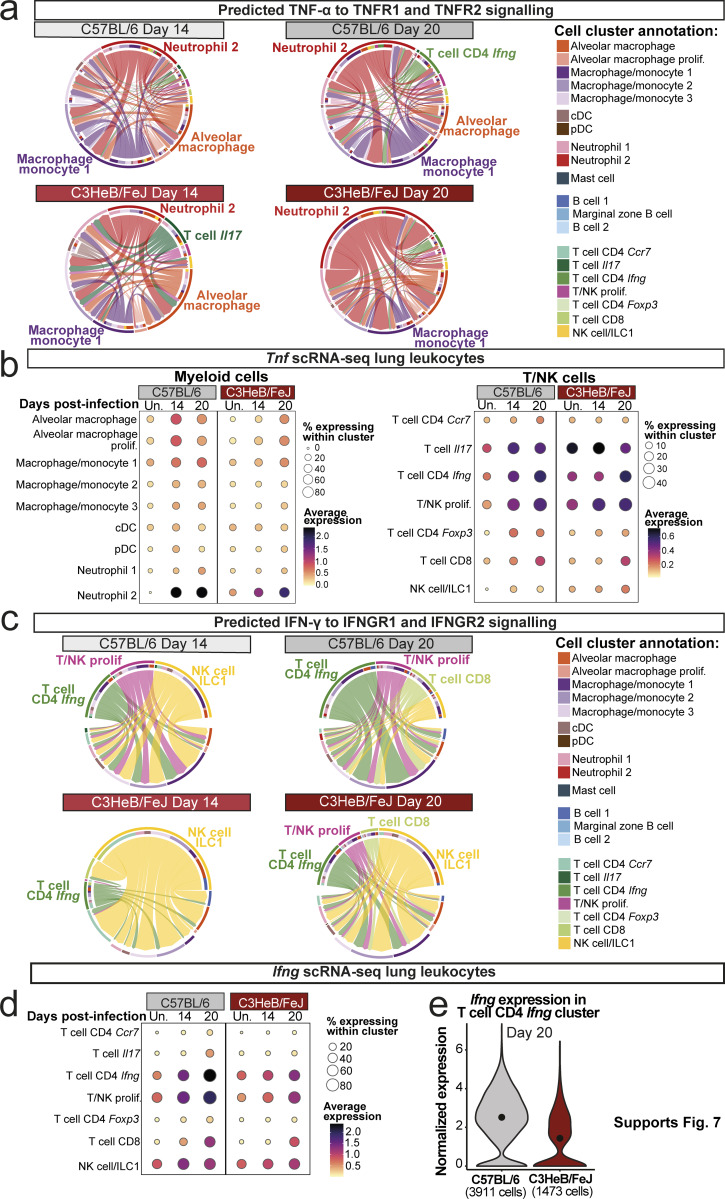
**Hallmark protective cytokines in lung leukocytes of C57BL/6 and C3HeB/FeJ mice. (a and c)** Chord plots showing the CellChat predicted relative interaction contributions of TNF-α and IFN-γ signaling to total interaction activity in each group. Outer circle and arrow colors indicate the predicted sending population, and the inner circle colors indicate the predicted receiving populations. Arrow thickness indicates the overall signaling contribution. **(b and d)** Dot plots showing expression of the indicated genes in scRNA-seq data. Circle sizes represent the abundance of cells expressing the gene, as a percentage of cells within the cluster. Circle color is proportional to the mean expression of the gene within all cells in the cluster. **(e)** Violin plot showing expression of *Ifng* within all cells in the T cell CD4 *Ifng* cluster at 20 days after infection, with a dot at the mean. Data shown are from a single scRNA-seq experiment, and plots show combined data from cells from *N* = 3 mice per group.

To identify possible pathways underpinning the earlier and more pronounced protective immune response in C57BL/6 mice, we examined known protective cytokine pathways in our bulk and scRNA-seq data. Our CellChat pathway analysis predicted much stronger TNF pathway signaling at 14 days after infection in C57BL/6 compared with C3HeB/FeJ mice, with comparable signaling strength predicted in the two strains by day 20 (Data S1 a). Accordingly, *Tnf* expression in whole lung was increased earlier in C57BL/6 mice but reached similar levels by 3 wk after infection, a pattern also observed for the protective cytokine *Il1b* (Fig. 7 a). However, predicted sources of TNF differed greatly between mouse strains, with the pro-inflammatory neutrophil 2 cluster dominating in C3HeB/FeJ mice, whereas *Tnf* expression in C57BL/6 mice derived from a combination of neutrophil 2, macrophage/monocyte 1, and T cell CD4 *Ifng* effector T cells (Fig. 7 b; and Fig. S4, a and b). Thus, TNF-α signaling likely operates distinctly in the contexts of protection and pathogenesis.

Significantly higher activity of the type II IFN (IFN-γ) pathway was inferred from scRNA-seq data in C57BL/6 than C3HeB/FeJ mice from 14 days after infection (Data S1 a). This was reflected in *Ifng* gene expression in lung tissue, which was increased at day 14 in C57BL/6, but not C3HeB/FeJ, mice and remained higher in C57BL/6 even at 21 days after infection (Fig. 7 a). Effector CD4 T cell populations expressing IFN-γ accumulated at day 20 in C57BL/6 (Fig. 3 d, Fig. S1, j–l, Fig. 7 b, and Fig. S4 d) and were predicted to provide IFN-γ signaling predominantly to the macrophage/monocyte 1 and 2 clusters (Fig. S4 c). Average expression of *Ifng* within the T cell CD4 *Ifng* cluster was also superior in C57BL/6 compared with C3HeB/FeJ mice (Fig. S4 e). We observed only very low expression of *Il17a* mRNA in lungs in all conditions analyzed, with modest induction observed at day 21 only in C57BL/6 mice (Fig. 7 a).

**Figure S5.**
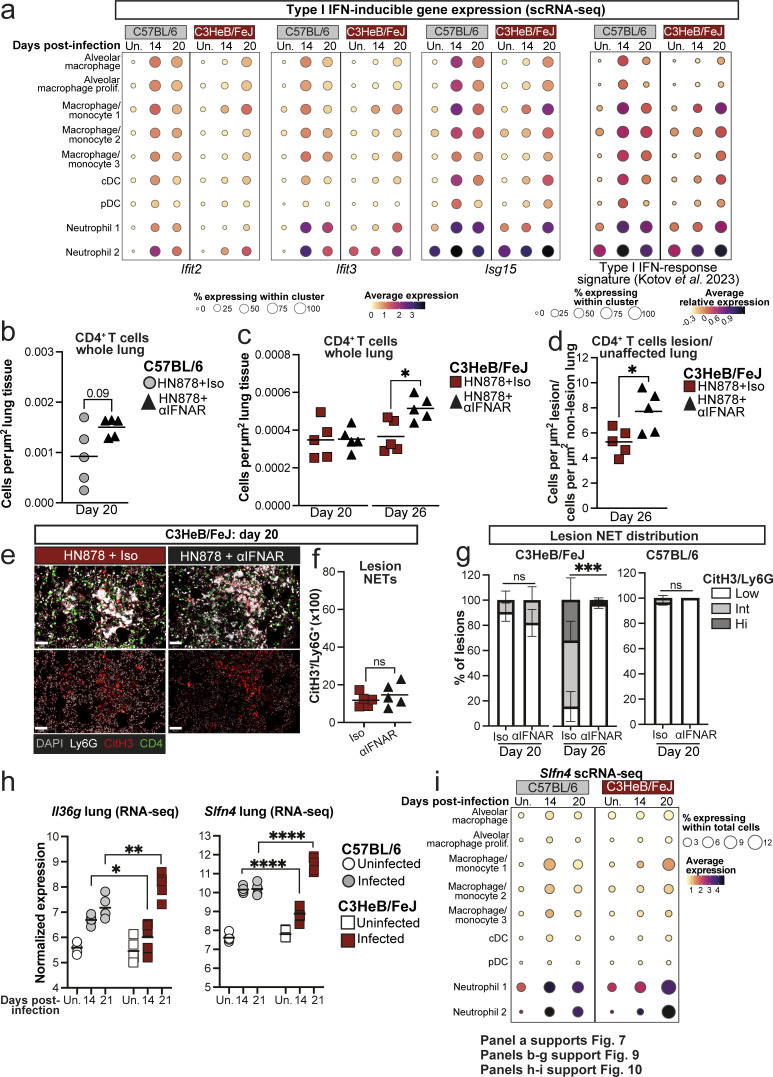
**Type I IFN-inducible gene expression and NETs in lungs of C57BL/6 and C3HeB/FeJ mice. (a)** Dot plots showing expression of individual representative ISGs or a 37-gene type I IFN-response signature (Kotov et al., 2023) in myeloid cell populations in scRNA-seq data. **(b–g)** Analysis of lung multiplex immunofluorescence in HN878-infected mice with and without IFNAR blockade in the experiments described in Figs. 8 and 9. **(b and c)** Numbers of total CD4^+^ T cells in whole left lungs normalized for tissue area. **(d)** Abundance of CD4^+^ T cells in lung lesions expressed relative to that in non-lesional lung. Statistical analysis: unpaired *t* test, with Welch’s correction applied in b. **(e)** Representative images showing all merged channels for NET staining (top) and CitH3 and DAPI alone (bottom) in C3HeB/FeJ mice at 20 days after infection. Scale bar = 50 μm. **(f)** Quantification of CitH3 NET staining relative to Ly6G staining in lung lesions. Statistical analysis: unpaired *t* test. **(g)** Percentage of lung lesions with low (CitH3/Ly6G < 0.2), intermediate (CitH3/Ly6G 0.2–0.4), or high (CitH3/Ly6G > 0.4) NET burden. Data shown are means ± standard error. Statistical analysis shown is Dirichlet-multinomial regression analysis of the effect of αIFNAR treatment on lesion NET status. Symbols indicate significant differences in the proportion of NET-low lesions. Data in b–g are from single experiments with *N* = 5 mice per group and are representative of two independent experiments. **(h)** DESeq2-normalized expression values of genes of interest as identified in Fig. 10 in whole lungs early in infection with HN878. Data shown are from a single bulk RNA-seq experiment with *N* = 5 mice per group and represent individual replicate mice as points with lines at the mean. Statistical analysis: two-way ANOVA with Holm–Sidak post hoc test. **(i)** Dot plot showing expression of *Slfn4* in scRNA-seq clusters. Data in a and i are from a single scRNA-seq experiment, and plots show combined data from cells from *N* = 3 mice per group. Circle sizes represent the abundance of cells expressing the gene, as a percentage of either cells in the cluster (a) or within total cells (i). Circle color is proportional to the mean expression of the gene within all cells in the cluster. Actual P values are shown or: *, P < 0.05; **, P < 0.01; ***, P < 0.001; ****, P < 0.0001; ns, not significant.

Unexpectedly, we also observed greater expression of ISGs in C57BL/6 mice compared with C3HeB/FeJ mice at 14 days after infection, with a limited further increase by day 21, by which point expression in C3HeB/FeJ had increased sharply to reach comparable levels (Fig. 7 c). ISG expression was observed widely across myeloid cell populations (Fig. 7 d and Fig. S5 a). Higher early expression in C57BL/6 mice was largely due to expression by MDMs and neutrophils, with these populations also representing the dominant ISG-expressing cells in TB-susceptible C3HeB/FeJ mice but only at the later day 20 time point (Fig. 7 d and Fig. S5 a). At this time point, ISG expression in C3HeB/FeJ mice was particularly pronounced in the pro-inflammatory neutrophil 2 cluster (Fig. 7 d and Fig. S5 a). Thus, in contrast to the later, high and sustained type I IFN response observed in C3HeB/FeJ mice (Moreira-Teixeira et al., 2020b), relatively TB-resistant C57BL/6 mice display a higher early ISG response that plateaus around the time of increased accumulation of effector T cells in the lung at 3 wk after infection.

### Type I IFN signaling limits early *M. tuberculosis* control in both C57BL/6 and C3HeB/FeJ mice, although effects in C57BL/6 mice wane at later time points

**Figure 8.**
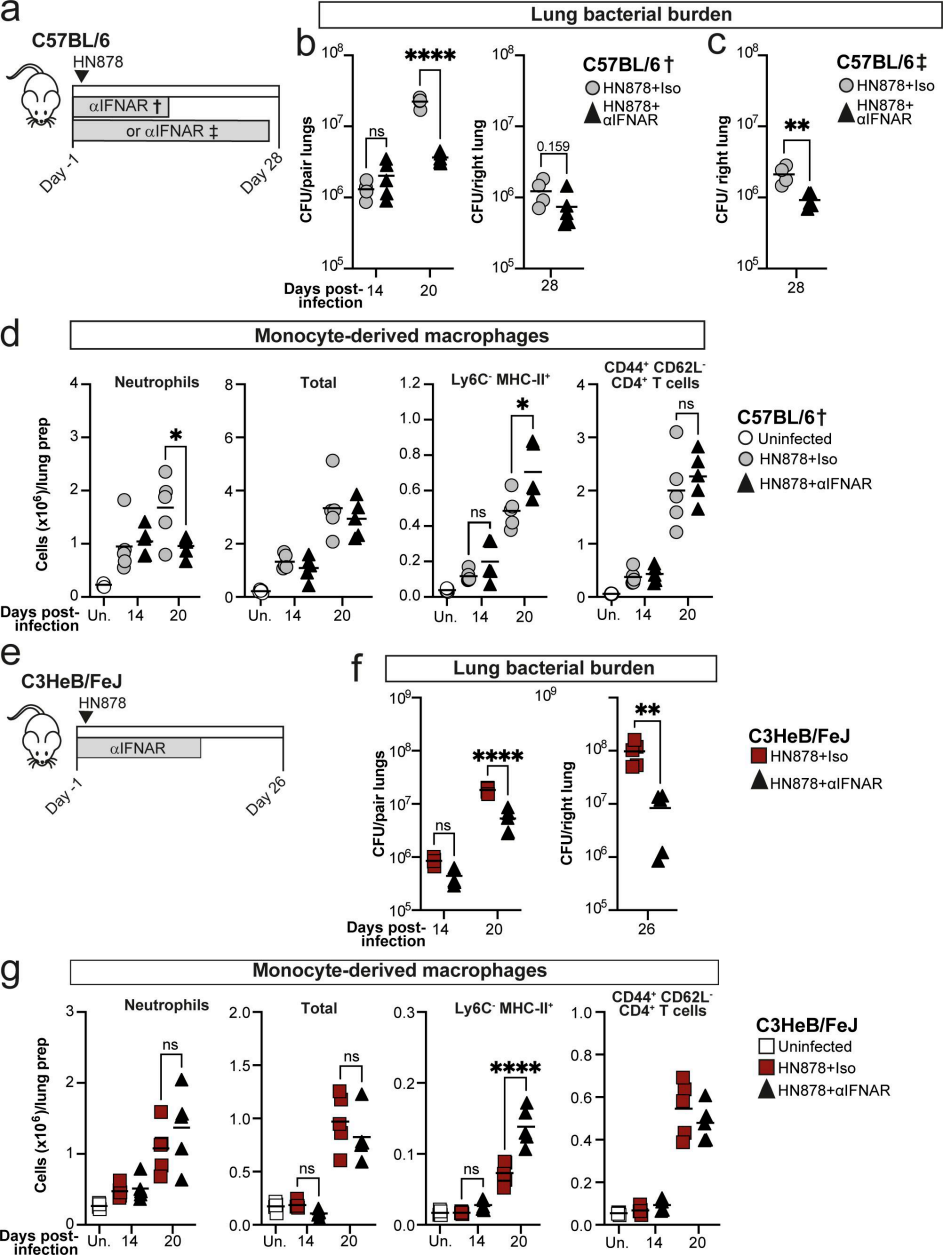
**Type I IFN signaling impairs early *M. tuberculosis* control in both C57BL/6 and highly TB-susceptible C3HeB/FeJ mice. (a)** C57BL/6 mice were aerosol infected with *M. tuberculosis* HN878 and received intraperitoneal injection of either anti-IFNAR (αIFNAR) or isotype control three times per week either between days −1 and 13 (†) or days −1 and 27 (‡). **(b and c)** CFU counts in lung tissue from either the (b) early (†) or (c) continuous (‡) αIFNAR treatment regimen. **(d)** Numbers of neutrophils (Ly6G^hi^CD11b^hi^CD45^+^), total and Ly6C^−^MHC-II^+^ MDMs (Siglec F^−^ Ly6G^−^ CD11b^+^CD64^+^MerTK^+^CD45^+^), and CD44^+^ CD62^−^ CD4^+^ T cells (CD3ε^+^CD45^+^) in lung tissue, as determined by flow cytometry. **(e)** C3HeB/FeJ mice were aerosol infected with *M. tuberculosis* HN878 and received intraperitoneal injection of either αIFNAR or isotype control three times per week between days −1 and 18. **(f)** CFU counts in lung tissue. **(g)** Numbers of neutrophils, total and Ly6C^−^ MHC-II^+^ MDMs, and CD44^+^CD62^−^ CD4^+^ T cells in lung tissue, as determined by flow cytometry. Points represent individual replicate mice with lines at the mean. Data are from single experiments with *N* = 4–5 mice per group and are representative of two independent experiments. Statistical analysis for CFU counts at day 26–28: unpaired *t* test. All other statistical analysis: two-way ANOVA with Holm–Sidak post hoc test. Actual adjusted P values are shown or: *, P < 0.05; **, P < 0.01; ****, P < 0.0001; ns, not significant.

We questioned whether the early lung type I IFN signaling observed in C57BL/6 mice, preceding substantial accumulation of CD4^+^ T cells in the lung, could contribute to disease progression, as in susceptible mice (Ji et al., 2019; Moreira-Teixeira et al., 2020a), or to initiation of a protective immune response to *M. tuberculosis*. Supporting a potential protective role, the type I IFN-inducible gene *Isg15*, which showed much greater induction of expression in C57BL/6 than C3HeB/FeJ mice at 14 days after infection (Fig. 7, c and d; and Fig. S5 a), has been implicated in promoting the protective IFN-γ response to mycobacteria (Bogunovic et al., 2012), and type I IFN signaling offers protection against *M. tuberculosis* in mice lacking IFN-γ signaling (Moreira-Teixeira et al., 2016). To test whether type I IFN contributed to early disease progression or protection in C57BL/6 mice, we performed transient IFNAR antibody blockade in C57BL/6 mice over the first 2 wk of infection († in Fig. 8 a). Surprisingly, early IFNAR blockade was protective in C57BL/6 mice, substantially reducing lung bacterial loads at 20 days after infection (Fig. 8 b). This protective effect was time point dependent, since a much more modest reduction of lung CFU was apparent at 28 days after infection (Fig. 8 b), even after blocking IFNAR throughout the infection (‡ in Fig. 8, a and c). Early IFNAR blockade resulted in a reduction in lung neutrophil numbers in C57BL/6 mice but did not impact total numbers of MDMs or CD44^+^CD62L^−^CD4^+^ T cells in lungs (Fig. 8 d). However, the Ly6C^−^MHC-II^+^ subpopulation of MDMs was specifically enriched in the anti-IFNAR-treated C57BL/6 mice at 20 days after infection (Fig. 8 d), suggestive of increased maturation and activation of lung MDMs, when early type I IFN signaling is blocked.

We previously reported that continuous IFNAR blockade of TB-susceptible C3HeB/FeJ mice offers partial protection at the peak of disease at 26 days after infection (Moreira-Teixeira et al., 2020a). However, whether type I IFN signaling contributes to susceptibility of these mice at earlier time points was unknown. We therefore performed IFNAR blockade up to 18 days after infection in C3HeB/FeJ mice (Fig. 8 e). Early IFNAR blockade was sufficient to reduce lung bacterial burden at 20 days after infection in C3HeB/FeJ mice, and protection was even more pronounced at day 26 (Fig. 8 f), similar to previous observations when anti-IFNAR was administered throughout infection (Moreira-Teixeira et al., 2020a). Early IFNAR blockade did not significantly affect total numbers of neutrophils, total MDMs, or CD44^+^CD62L^−^CD4^+^ T cells in lungs, but Ly6C^−^MHC-II^+^ MDM numbers were increased at 20 days after infection (Fig. 8 g), mirroring results in C57BL/6 mice.

Overall, we unexpectedly observed that early type I IFN signaling early during *M. tuberculosis* infection contributed to disease progression in relatively resistant C57BL/6 mice as well as in highly TB-susceptible mice, although C57BL/6 mice overcome this later in infection as the CD4^+^ T cell response increases. However, more pronounced, sustained, detrimental effects of type I IFN signaling are observed at later time points in C3HeB/FeJ mice than in C57BL/6 mice.

### Type I IFN signaling promotes neutrophil swarming and restricts CD4^+^ T cell accumulation in TB lesions of both relatively resistant and highly TB-susceptible mice

**Figure 9.**
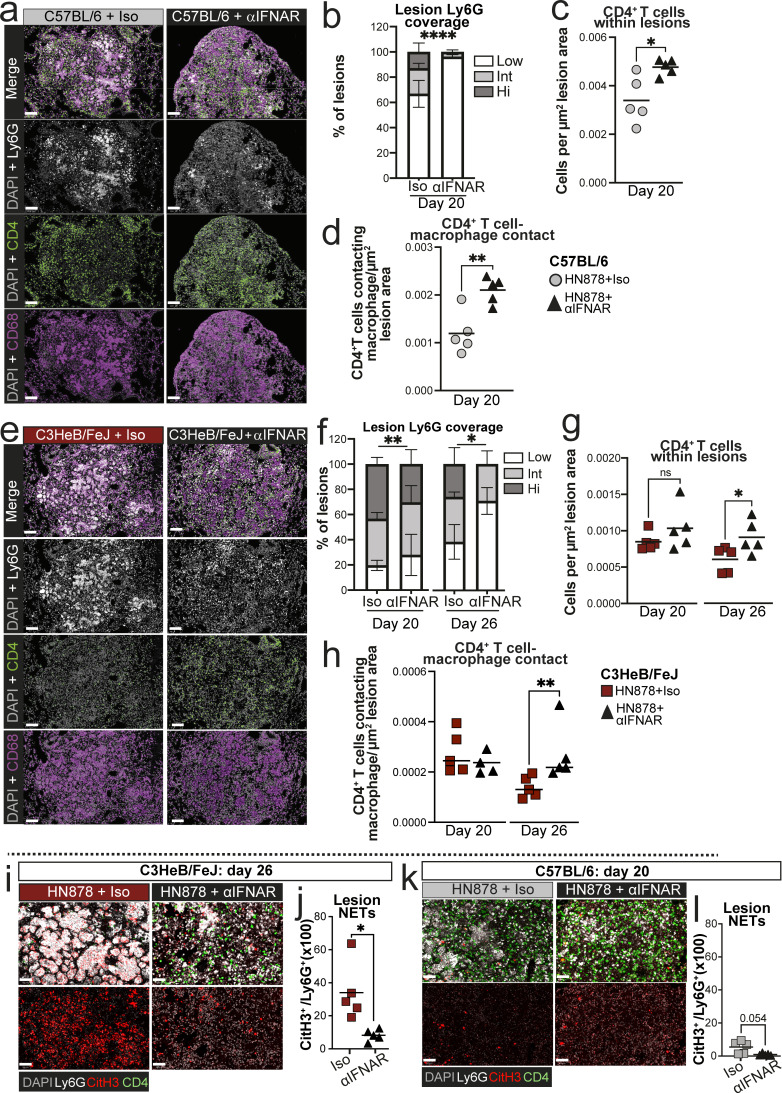
**Early type I IFN signaling promotes neutrophil swarming and limits CD4** ^**+**^ **T cell numbers in TB lesions of both relatively resistant and highly TB-susceptible mice.** Lung sections from experiments described in Fig. 8 were analyzed by multiparameter immunofluorescence. **(a)** Images of representative lesions from C57BL/6 mice treated with either early anti-IFNAR (αIFNAR) or isotype control, at 20 days after infection. Individual fluorescent channels and merged images are shown. **(b)** Stacked bar plots showing percentages of lesions across whole left lungs falling into low (≤20%), intermediate (Int, >20% ≤40%), or high (Hi, >40) bins for coverage with Ly6G staining. **(c and d)** Numbers of total CD4^+^ T cells (c) and CD4^+^ T cells in contact with a macrophage annotation (≤0 μm distance, d) within lung lesions at 20 days after infection, normalized for the total area of all lesions across whole left lungs. **(e)** Images of representative lesions from C3HeB/FeJ mice treated with either early anti-IFNAR (αIFNAR) or isotype control at 26 days after infection. Individual fluorescent channels and merged images are shown. **(f)** Stacked bar plots showing percentages of lesions across whole left lungs falling into low (≤20%), intermediate (Int, >20% ≤40%), or high (Hi, >40) bins for coverage with Ly6G staining. **(g and h)** Number of total CD4^+^ T cells (g) and CD4^+^ T cells in contact with a macrophage annotation (≤0 μm distance, h) within lung lesions at the indicated time points, normalized for the total area of all lesions across whole left lungs. **(i–l)** Immunofluorescence staining for CitH3 and Ly6G to detect NETs in lung lesions at the indicated time points. **(i and k)** Representative images showing all merged channels (top) or CitH3 and DAPI alone (bottom) in C3HeB/FeJ (i) and C57BL/6 (k) mice. **(j and l)** Quantification of CitH3 NET staining relative to Ly6G staining in lung lesions. Data shown in b and f are means ± standard error of *N* = 5 per group. Statistical analysis shown in b and f is Dirichlet-multinomial regression analysis of the effect of αIFNAR treatment on lesion composition. Symbols indicate significant differences in the proportion of Ly6G-low lesions. Plots in c, d, g, h, j, and l show individual replicate mice as points with lines at the mean or median (h). Statistical analysis in b and g: unpaired *t* test. Statistical analysis in h: Mann–Whitney test. Statistical analysis in j and l: unpaired *t* test with Welch’s correction. Actual P values are shown or: *, P < 0.05; **, P < 0.01; ****, P < 0.0001; ns, not significant. Data shown are from single experiments with *N* = 5 mice per group that are representative of two independent experiments. Scale bars in a and e = 100 μm; scale bars in i and k = 50 μm. See also: Fig. S5, b–g.

Given that early type I IFN signaling favored *M. tuberculosis* replication and restricted accumulation of Ly6C^−^MHC-II^+^ MDMs and CD4^+^ T cell infiltration of TB lesions, we asked whether early IFNAR blockade allowed greater CD4^+^ T cell infiltration of TB lesions that could facilitate protective T cell–macrophage interactions and MDM activation. Although most TB lesions in C57BL/6 mice at 20 days after infection had low Ly6G coverage, this was further increased to nearly 100% of lesions by early IFNAR blockade, with very few areas of continuous Ly6G staining apparent (Fig. 9, a and b). This is consistent with neutrophil swarming in early lesions being type I IFN dependent in these mice. Conversely, the abundance of CD4^+^ T cells within lesions was increased in anti-IFNAR–treated mice, resulting in a greater number of CD4^+^ T cell–macrophage interactions (Fig. 9, a, c, and d). Generally higher CD4^+^ T cell numbers were also observed across whole lung tissue in anti-IFNAR–treated infected C57BL/6 mice compared with those receiving isotype control, but this effect was less pronounced than in the lesions (Fig. S5 b).

Applying this analysis to highly susceptible C3HeB/FeJ mice, we found early anti-IFNAR treatment to increase the proportion of Ly6G^low^ lesions from 20 days after infection, with a more marked effect at the peak of disease at day 26 (Fig. 9, e and f). Abundance of CD4^+^ T cells and CD4^+^ T cell–macrophage contacts within lesions was also increased at 26 days after infection in anti-IFNAR–treated C3HeB/FeJ mice (Fig. 9, g and h), concurrent with the high degree of protection observed at this time point (Fig. 8 f). Total lung CD4^+^ T cells were also increased in anti-IFNAR–treated C3HeB/FeJ at day 26 (Fig. S5 c); however, the magnitude of increased CD4^+^ T cell abundance in lesions exceeded that observed in non-lesional lung tissue (Fig. S5 d), suggesting that the effect of anti-IFNAR on CD4^+^ T cell accumulation is most pronounced in the lesions.

Having previously published that type I IFN drives NET formation in TB-susceptible mice at the peak of disease (Moreira-Teixeira et al., 2020a), we reasoned that type I IFN-dependent neutrophil NETosis could impede T cell access to infected macrophages. Quantification of NETs by immunofluorescence staining of citrullinated histone H3 (CitH3), alongside immune cell markers, confirmed our previous findings (Moreira-Teixeira et al., 2020a) that C3HeB/FeJ mice display extensive NET accumulation in lung lesions, which was largely ablated by early IFNAR blockade at 26 days after infection, with minimal effects at day 20 after infection (Fig. 9, i and j; and Fig. S5, e–g). In contrast, minimal NET accumulation was detected in lesions of C57BL/6 mice at day 20 after infection (Fig. 9, k and l; and Fig. S5 g), despite the marked effects of IFNAR blockade on lesion Ly6G coverage and CD4^+^ T cell accumulation at this time point in C57BL/6 mice (Fig. 9, a–d). These data indicate that NETosis cannot be the sole mechanism of type I IFN restriction of CD4^+^ T cell accumulation in TB lesions.

Overall, we show that blockade of early type I IFN signaling increases the ratio of CD4^+^ T cells to neutrophils in TB lesions of both C57BL/6 and C3HeB/FeJ mice, suggesting that early induction of type I IFN signaling during *M. tuberculosis* infection acts to favor neutrophil accumulation and limit CD4^+^ T cell infiltration into developing granulomatous lesions, with the timing and magnitude of this common mechanism differing on susceptible and resistant backgrounds.

### Gene expression signatures of severe TB in C3HeB/FeJ mice are partially ameliorated by type I IFN receptor blockade

**Figure 10.**
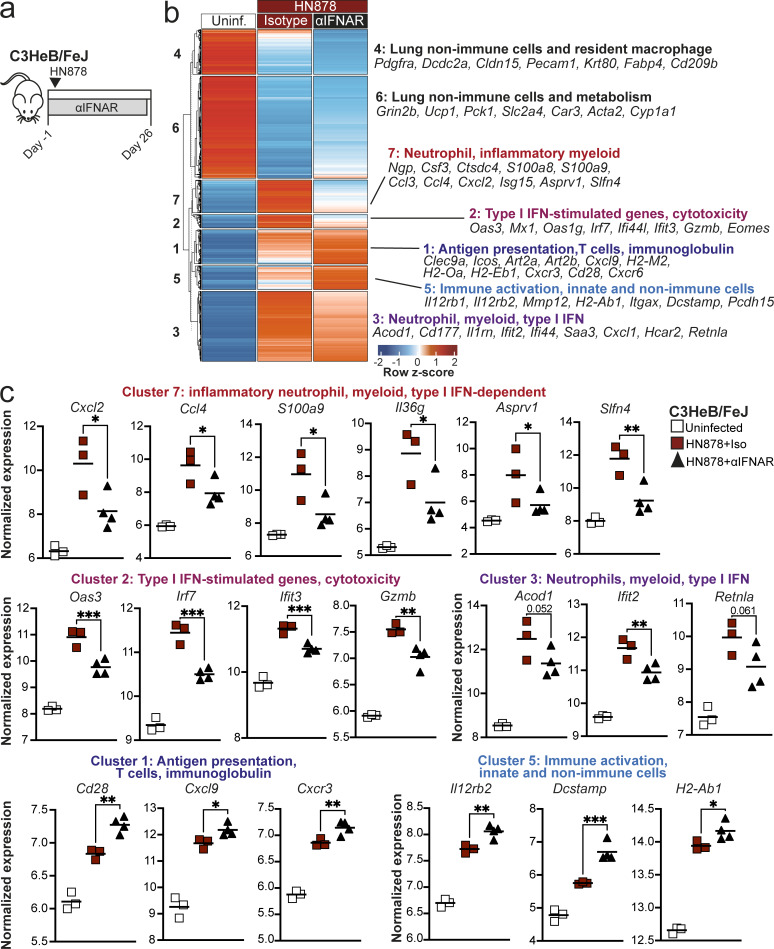
**Type I IFN blockade abrogates the inflammatory neutrophil gene signature in TB-susceptible mice and increases expression of genes associated with a T cell response.** Bulk RNA-seq was performed on lung tissue from C3HeB/FeJ mice aerosol infected with HN878 while being treated with either anti-IFNAR (αIFNAR, *N* = 4) or isotype control (*N* = 3) three times per week from day −1 to day 25, as compared with uninfected mice receiving isotype control antibody (*N* = 3). **(a)** Experimental scheme outline. Analysis was performed at the peak of disease at 26 days after infection. **(b)** All DEGs in either infected group compared with uninfected controls were subjected to k-means clustering. Clusters are annotated based on hallmark genes and pathways. **(c)** DESeq2-normalized expression values of representative genes from k-means clusters. Data shown represent individual replicate mice as points with lines at the mean. Data are from a single bulk RNA-seq experiment. Statistical analysis: two-way ANOVA with Holm–Sidak post hoc test. Actual adjusted P values are shown or: *, P < 0.05; **, P < 0.01; ***, P < 0.001. See also: Fig. S5, h and i.

Finally, we examined the wider impact of the later, sustained type I IFN response in TB-susceptible C3HeB/FeJ mice on the lung gene expression signature observed at the peak of disease by RNA-seq of lung tissue at 26 days after infection, with and without continuous IFNAR blockade (Fig. 10 a). Clustering analysis of differentially expressed genes (DEGs) revealed a reduction in expression of genes relating to nonimmune cell function upon infection without any effects of IFNAR blockade (Fig. 10 b, clusters 4 and 6), likely reflecting loss of steady-state lung structure and function resulting from *M. tuberculosis* infection. Of the clusters that increased during infection, cluster 3 contained subsets of type I IFN-inducible and inflammatory myeloid-related genes that were only modestly reduced by anti-IFNAR treatment, including *Acod1*, *Ifit2*, and *Retnla* (Fig. 10, b and c). In contrast, two clusters were most greatly diminished in expression in anti-IFNAR–treated compared with infected control mice (Fig. 10, b and c, clusters 2 and 7). One of these clusters was dominated by ISGs, such as *Oas3*, *Irf7*, and *Ifit3*, as well as genes related to cytotoxic lymphocyte function, such as *Gzmb* (Fig. 10, b and c, cluster 2). The other included a large subset of neutrophil-related genes, including the chemokines *Cxcl2*, *Ccl3*, and *Ccl4* (Fig. 10, b and c, cluster 7), which we had determined to be enriched in the pro-inflammatory neutrophil 2 cluster from our scRNA-seq data that increased with progressive disease in C3HeB/FeJ mice (Fig. 3, b and c; and Fig. S1, j–l), consistent with exacerbation of neutrophil activation by type I IFNs. Deeper interrogation of this cluster also highlighted previously unidentified type I IFN−dependent genes in TB-susceptible C3HeB/FeJ mice, including *Il36g*, encoding IL-36*γ*, a cytokine implicated in mucosal inflammatory responses (Yuan et al., 2019), and *Slfn4*, encoding Schlafen 4 (Fig. 10, b and c), previously identified as a marker of myeloid cells with immune-suppressive function in a gastric metaplasia model (Ding et al., 2016). Consistent with an association of these genes with progression toward severe TB, we observed a more pronounced increase in *Il36g* and *Slfn4* expression in lungs of C3HeB/FeJ compared with C57BL/6 mice at 21 days after infection, and *Slfn4* expression was most highly enriched in the disease-associated, pro-inflammatory neutrophil 2 scRNA-seq cluster (Fig. S5, h and i).

We conversely observed increased expression of genes related to antigen presentation and T cell responses, including *H2-Ab1*, *Cd28*, *Cxcl9*, *Cxcr3*, and *Il12rb2* with IFNAR blockade compared with control-infected mice (Fig. 10, b and c, clusters 1 and 5). These findings suggest that type I IFN signaling contributes substantially to the pro-inflammatory neutrophil response in TB-susceptible C3HeB/FeJ mice, including the elevation of pro-inflammatory chemokines *Ccl3*, *Ccl4*, and *Cxcl2*, as well as limited expression of the effector T cell–attractant chemokine *Cxcl9* observed during disease progression in these mice, in accordance with the improved CD4^+^ T cell accumulation in TB lesions observed with IFNAR blockade.

## Discussion

Immune cells and cytokines are likely to have context-dependent functions at different stages of *M. tuberculosis* infection or in hosts differing in their genetic susceptibility to TB or presence of comorbidities. Laboratory mouse strains markedly differ in their susceptibility to TB (Kramnik and Beamer, 2016; Meade and Smith, 2025), with more resistant strains allowing dissection of protective immune pathways and highly susceptible strains offering models of pathogenesis relevant to human disease. While we have previously characterized the local and systemic immune response in resistant and susceptible mouse strains during established TB disease (Moreira-Teixeira et al., 2020b), the early stages of infection preceding the distinct outcomes in these mouse models have not been described. Here, we combined bulk and scRNA-seq with flow cytometry, spatial immunofluorescence analysis, and in vivo cell and cytokine disruption to dissect the early immune response to *M. tuberculosis* infection in relatively TB-resistant C57BL/6 mice and highly TB-susceptible C3HeB/FeJ mice. We observed a more pronounced early immune response in C57BL/6 compared with C3HeB/FeJ mice, which was accompanied by a higher early pulmonary type I IFN response. We demonstrate that early type I IFN signaling drives common pathogenic mechanisms in C57BL/6 as well as C3HeB/FeJ mice, but that major sustained pathogenic effects are limited to TB-susceptible mice.

Our observation of higher expression of ISGs early in *M. tuberculosis* infection in C57BL/6 than C3HeB/FeJ mice was unexpected, given that high and sustained type I IFN responses are known to contribute to susceptibility of C3HeB/FeJ mice and in C57BL/6 mice bearing the C3HeB/FeJ *Sst1*^*s*^ susceptibility locus at later time points during established disease (Ji et al., 2019; Moreira-Teixeira et al., 2020a). Moreover, blood transcriptomics work has strongly implicated type I IFN signaling in human TB pathogenesis and progression (Berry et al., 2010; Scriba et al., 2017; Tabone et al., 2021; Zak et al., 2016). We considered that the early spike in type I IFN signaling in C57BL/6 mice could contribute to host protection, as had been suggested by some previous reports (Bogunovic et al., 2012; Moreira-Teixeira et al., 2016), and since type I IFN can activate various immune cells important for infection control (McNab et al., 2015; Moreira-Teixeira et al., 2018). However, early IFNAR blockade unexpectedly showed similar protective effects on bacterial load, neutrophil swarming, and lesion CD4^+^ T cell–macrophage interactions in both resistant and susceptible mouse strains.

These common pathogenic effects differed principally in their timing. We observed the most pronounced protective effect of early IFNAR blockade in C57BL/6 mice at 20 days after infection, when the lung CD4^+^ T cell response is still in its early stages, with only modest reductions in lung CFU observed when analyzed at the later 28-day time point, the latter in keeping with past reports (Ji et al., 2019; Mayer-Barber et al., 2011; Moreira-Teixeira et al., 2017; Moreira-Teixeira et al., 2016). Conversely, in C3HeB/FeJ mice, which we show to mount a delayed and limited CD4^+^ T cell response to *M. tuberculosis* infection, protective effects of early IFNAR blockade were maintained at the peak of disease, consistent with our previously reported study using continuous IFNAR blockade (Moreira-Teixeira et al., 2020a). Further study will be required to determine whether the observed effects of IFNAR blockade during HN878 infection also occur with less virulent *M. tuberculosis* strains. Collectively, these data support a model in which the protective effector CD4^+^ T cell response mounted in C57BL/6 mice helps overcome disease progression in lung lesions, limiting the pathogenic effects of type I IFN. In contrast, delayed early immune activation, together with the defect in type I IFN regulation conferred by the *Sst1*^*s*^ locus (Ji et al., 2019), allows sustained pathogenic effects of type I IFNs in C3HeB/FeJ mice.

How exactly type I IFN signaling promotes early *M. tuberculosis* infection in both resistant and susceptible mice remains to be elucidated. Our flow cytometry data suggest a role for impaired MDM maturation and activation that is ameliorated by IFNAR blockade. Accordingly, we observed strong transcriptional signatures of type I IFN response in MDMs of both C57BL/6 and C3HeB/FeJ mice that correlated with those detected in whole lungs, consistent with results from *Sp140*^−/−^ mice (Kotov et al., 2023). Our scRNA-seq analysis identified MDMs as major contributors to the highly distinct chemokine expression profiles of C57BL/6 and C3HeB/FeJ mice early in infection, and we additionally showed the excessive pro-inflammatory chemokine expression in C3HeB/FeJ mice to be type I IFN dependent, likely at least in part reflecting an effect on MDMs. Lung macrophages differ in their ability to control *M. tuberculosis* infection depending on both their ontogeny, activation state, and metabolism (Huang et al., 2018; Lai et al., 2024; Pisu et al., 2024; Zheng et al., 2024), and so cell-intrinsic effects of type I IFN signaling on the ability of MDMs to control infection are likely to contribute to susceptibility.

Excessive accumulation of neutrophils is associated with failed *M. tuberculosis* control in mouse models (Keller et al., 2006; Kimmey et al., 2015; Moreira-Teixeira et al., 2020a; Moreira-Teixeira et al., 2020b; Nandi and Behar, 2011) and advanced TB disease in humans (Condos et al., 1998). Our current study shows that neutrophils, particularly an inflammatory population expressing *Cxcl2*, *Ccl3*, *Ccl4*, and *Slfn4*, increase in line with the type I IFN response in both C57BL/6 and C3HeB/FeJ mice. These inflammatory neutrophils expressed high levels of ISGs, and their accumulation was more sustained and pronounced in C3HeB/FeJ mice. Expression of the signature genes of this population was diminished by IFNAR blockade in C3HeB/FeJ mice, accompanying reduced Ly6G coverage, suggestive of neutrophil swarming, in lesions of these mice. Collectively, these data support sustained, as opposed to transient, type I IFN activation of neutrophils as a driver of TB susceptibility in C3HeB/FeJ mice.

We observed increased pulmonary CD4^+^ T cell responses from early time points after infection in C57BL/6 mice compared with C3HeB/FeJ mice that were independent of the infecting *M. tuberculosis* strain. Our scRNA-seq analysis suggested more extensive early MDM–CD4^+^ T cell interactions in C57BL/6 than C3HeB/FeJ mice, including superior predicted signaling via the protective cytokines IFN-γ and TNF-α and increased expression of T cell–attractant chemokine genes, *Cxcl9* and *Cxcl10*, by MDMs. We did not observe substantial *Il17a* expression in lungs of mice at the early time points analyzed here, nor was the *Il17*-expressing T cell cluster observed in our scRNA-seq data differentially abundant in the two mouse strains during infection. IL-17 responses have been implicated both in protection and pathogenesis during *M. tuberculosis* infection. Peripheral blood Th17-like T cell phenotypes have been associated with latently infected individuals compared with those progressing to TB (Scriba et al., 2017), as well as individuals who “resist” *M. tuberculosis* infection without developing a peripheral IFN-γ response (Sun et al., 2024). Conversely, IL-17A is enriched in skin recall responses of active TB patients (Pollara et al., 2021). IL-17A has been shown to contribute to protection of C57BL/6 mice from HN878 infection from 30 days after infection (Gopal et al., 2014); however, our data suggest that it is unlikely to mediate the very early differences observed in the pulmonary immune response in our study. Natural killer cells and CD8α^+^ T cells have also been implicated in early protection against *M. tuberculosis* infection (Roy Chowdhury et al., 2018; Winchell et al., 2023) but did not show clear association with protection in our analysis.

Our data support CD4^+^ T cell abundance and frequency of CD4^+^ T cell–macrophage contacts in TB lesions as a correlate of the protection offered by IFNAR blockade in both resistant and susceptible mice; however, the mechanism linking early type I IFN signaling to limitation of CD4^+^ T cell numbers in lesions remains to be elucidated. A potential explanation is local inhibitory effects of lesion neutrophils on CD4^+^ T cells. We observe an inverse relationship between pulmonary neutrophils and CD4^+^ T cells during *M. tuberculosis* infection and found IFNAR blockade to limit neutrophil clustering and increase CD4^+^ accumulation in lesions of both C57BL/6 and C3HeB/FeJ mice. Further studies will be required to determine how neutrophils could restrict CD4^+^ T cell accumulation in TB lesions. One possibility is direct immunosuppressive function of neutrophils. We observed high, type I IFN–dependent, expression of *Slfn4* in lungs of C3HeB/FeJ mice and found this gene to be particularly enriched in the pro-inflammatory, neutrophil population that increased with disease progression in these mice. *Slfn4* is also highly expressed during severe TB in *Nos2*^−/−^ mice (Beisiegel et al., 2009) and is a marker gene for suppressive myeloid cells in gastric metaplasia (Ding et al., 2016). Whether *Slfn4*-expressing neutrophils have immunosuppressive function in TB remains to be determined. Type I IFN–induced NET formation is associated with TB susceptibility of C3HeB/FeJ mice at the peak of disease (Moreira-Teixeira et al., 2020a) and has been recently reported to promote *M. tuberculosis* replication in neutrophils in vitro (Sur Chowdhury et al., 2024). NETs induced by CXCR1/2 agonists have been shown to impede cytotoxic lymphocyte interactions with tumor cells in mouse cancer models and in vitro systems (Teijeira et al., 2020). However, we only observed type IFN-dependent NET accumulation in C3HeB/FeJ mice and not in C57BL/6 mice, indicating that NETs cannot solely explain the type I IFN–activated neutrophil interference with CD4^+^ T cell–macrophage interactions in developing TB lesions.

Increased lesion CD4^+^ T cell numbers are not sufficient to explain the reduced lung *M. tuberculosis* burdens resulting from IFNAR blockade in our models, since we observed a significant reduction in lung bacterial burden in C3HeB/FeJ mice before any increase in CD4^+^ T cells upon IFNAR blockade. Indeed, type I IFN is likely to operate via several interacting pathogenic mechanisms, including macrophage necrosis (Dorhoi et al., 2014; Zhang et al., 2021), recruitment of permissive mononuclear phagocytes (Antonelli et al., 2010), NET production (Moreira-Teixeira et al., 2020a; Sur Chowdhury et al., 2024), and suppression of protective IL-1 signaling (Ji et al., 2019; Mayer-Barber et al., 2014). Nonetheless, increased CD4^+^ T cell–macrophage interactions following IFNAR blockade likely contribute to sustained protection. Diminished IFN-γ response signatures and IFNGR expression have been demonstrated in MDMs with a high type I IFN response signature in *M. tuberculosis*–infected *Sp140*^−/−^ mice (Kotov et al., 2023). Although this could partially reflect cell intrinsic cross-regulation of type I and II IFNs, our data suggest that limited CD4^+^ T cell–macrophage cross talk due to distinct lesion organization also contributes to limited IFN-γ responses in macrophages and impaired *M. tuberculosis* control in the context of type I IFN–dependent TB susceptibility.

IFNAR blockade did not completely protect C57BL/6 and C3HeB/FeJ mice, consistent with there being type I IFN-dependent and -independent components to TB susceptibility of these mice. Indeed, although the C3HeB/FeJ *Sst1*^s^ allele is sufficient to confer type I IFN–dependent TB susceptibility in C57BL/6 mice (Ji et al., 2019), these mice still remain less susceptible than C3HeB/FeJ (Pichugin et al., 2009). The delayed early MDM and CD4^+^ T cell response that we report in C3HeB/FeJ mice was not corrected by IFNAR blockade, suggesting that this delay is type I IFN independent. This is in contrast to the recently reported TB susceptibility phenotype in C57BL/6 mice carrying a deletion in the IFN-γ–inducible GTPase gene *Irgm1* (Rai et al., 2023), which display impaired CD4^+^ T cell response initiation dependent on excessive type I IFN production (Naik et al., 2024). We did not observe deficiency in *Irgm1* expression in lungs of C3HeB/FeJ mice (data available at: https://ogarra.shinyapps.io/earlymousetb/).

The delayed and limited CD4^+^ T cell response in C3HeB/FeJ mice is likely due in part to differences in MHC alleles from C57BL/6 (Kramnik and Beamer, 2016). While delayed dissemination of *M. tuberculosis* Erdman strain to lung-draining lymph nodes has been reported in C3H/HeJ mice (Chackerian et al., 2002), this was not apparent in our experiments where C3HeB/FeJ mice were infected with the *M. tuberculosis* strain HN878. It remains possible that the increased early *M. tuberculosis* bacterial load observed at 2 wk after infection in C57BL/6 compared with C3HeB/FeJ mice drives the earlier MDM and T cell responses in C57BL/6 mice, although the interplay between very early bacterial load and immune response initiation is challenging to unpick. Early lung macrophage dynamics have been reported to affect T cell priming following infection of C3HeB/FeJ mice with particular clinical *M. tuberculosis* isolates (Lovey et al., 2022). Thus, mechanisms linking the limited early MDM and CD4^+^ T cell responses in C3HeB/FeJ mice warrant further investigation. Our scRNA-seq analysis highlighted intrinsic differences in expression of notable ligand genes in macrophage populations between C3HeB/FeJ and C57BL/6 mice prior to and during infection, including higher expression of *Spp1* and *Siglec1* (CD169) by C3HeB/FeJ mice and higher expression of *Apoe* by several populations, particularly MDMs, in C57BL/6 mice. *Apoe*^−/−^ mice on a C57BL/6 background are hypersusceptible to TB when fed a high-cholesterol diet (Martens et al., 2008) and develop severe disease dependent on type I IFN signaling and ameliorated by an inhibitor of PAD4-dependent NETosis (Liu et al., 2025). Determining whether lung MDM-derived ApoE plays a role in protection against *M. tuberculosis* will require further study.

We were unable to use intravascular CD45 labeling prior to generation of single-cell suspensions, so we could not discriminate parenchymal leukocytes from those remaining in the lung vasculature after perfusion. This could impact the relative abundance of leukocyte subsets analyzed in single-cell suspensions between conditions, particularly if there is a difference in lung vascular leukocyte retention between C57BL/6 and C3HeB/FeJ mice, which is not known. However, our key findings from scRNA-seq and flow cytometry were validated by microscopy of lung lesions, which overcomes this limitation. Although it would have been of interest to precisely localize infected cells in our immunofluorescence analysis using *M. tuberculosis* fluorescent reporter strains, the signal at early time points following low-dose aerosol infection may limit this approach.

In conclusion, we characterized the early cellular interactions preceding initial control of *M. tuberculosis* infection in C57BL/6 mice as compared with failed immune control in highly TB-susceptible C3HeB/FeJ mice. We show that type IFN drives common early pathogenic effects in C57BL/6 and C3HeB/FeJ mice, increasing lung bacterial burden and limiting macrophage–CD4^+^ T cell interactions, with more pronounced later effects on inflammatory neutrophil activation observed in the context of high, sustained type I IFN signaling on a TB-susceptible genetic background.

## Materials and methods

### Mice and ethics

C57BL/6 and C3HeB/FeJ mice were either bred and housed in specific pathogen–free facilities at The Francis Crick Institute, London, UK (for *M. tuberculosis* HN878 infection experiments), or purchased from Charles River Laboratories and housed in specific pathogen–free facilities at Instituto de Investigação e Inovação em Saúde (i3S) (for *M. tuberculosis* 6C4 and 4I2 experiments). Experiments with HN878 were performed in the UK in accordance with Home Office (UK) requirements and the Animal Scientific Procedures Act, 1986, under the specific Project License PP3881464. Experiments with 6C4 and 4I2 were performed in Portugal with recommendations of the European Union Directive 2010/63/EU and approved by the Portuguese National Authority for Animal Health—Direção Geral de Alimentação e Veterinária (DGAV-Ref. #018413/2021-11-24). Mice were kept under specific pathogen–free conditions, at controlled temperature (20–24°C), humidity (45–65%), and light cycle (12 h light/dark). Mice were maintained with ad libitum access to food and water. Female mice were used for HN878 experiments. Male and female mice were used, in equal proportions, for 6C4 and 4I2 experiments. Mice were euthanized humanely by intraperitoneal injection with an overdose of anesthetic.

### *M. tuberculosis* strains

Three strains were used in this study: the highly virulent clinical isolate HN878 and additional clinical isolates determined to cause either severe (6C4 strain) or mild (4I2 strain) in humans (Sousa et al., 2020). Strains were grown to mid-logarithmic phases in Middlebrook 7H9 broth, supplemented with 10% Middlebrook oleic acid albumin dextrose complex (OADC, BD) 0.05% Tween-80 and 0.5% glycerol and stored in aliquots at −80°C.

### Experimental infections

Mice were infected via the aerosol route using an inhalation exposure system (Glas-Col), calibrated to deliver 150–350 (HN878 and 6C4 strains) or 500–1000 (4I2 strain) CFUs to the lungs of each mouse. *M. tuberculosis* uptake was confirmed in each experiment by euthanizing three to five mice immediately after infection and determining lung bacterial load. For HN878 experiments, C57BL/6 and C3HeB/FeJ mice were infected in separate infection runs due to space constraints. Comparable HN878 uptake was determined over multiple infections in C57BL/6 and C3HeB/FeJ mice, as detailed in Fig. S1. The confirmatory experiments using 6C4 and 4I2 strains at i3S, Porto, Portugal, were performed with all mice in the same infection run. Mice were euthanized at predetermined time points after infection, as indicated in the respective figures, by intraperitoneal injection with an overdose of anesthetic. A previously determined maximum endpoint of 26 days after infection was used for C3HeB/FeJ mice infected with HN878, since excessive clinical severity can occur after this point, while immune signatures and lung pathology at later time points remain comparable with those observed at day 26 (Moreira-Teixeira et al., 2020a; Moreira-Teixeira et al., 2020b). Age- and sex-matched uninfected control mice, housed in the same room but isolated from infected animals, were included in experiments.

### In vivo Neutrophil depletion and type I IFN receptor blockade

For neutrophil depletion, mice received 0.2 mg of rat anti-mouse Ly6G (clone 1A8; Bio X Cell) or rat IgG_2a_ isotype control (clone 2A3; Bio X Cell) in 0.2 ml volume of sterile PBS by intraperitoneal injection. Mice were injected three times per week starting on day 12 after infection, and treatment continued until day 25 after infection. For type I IFN receptor blockade, mice received 0.5 mg of mouse anti-mouse IFNAR (clone MAR1-5A3; Leinco Technologies) or mouse IgG_1_ isotype control (clone HKSP; Leinco Technologies) in 0.2 ml volume of sterile PBS by intraperitoneal injection. Mice were injected three times per week starting 1 day prior to infection, and treatment continued for the required duration, as indicated in the relevant figures.

### Organ bacterial burden quantification

Organs were collected into sterile PBS and disrupted by gently pressing through 70-μM cell strainers. Serial dilutions of organ homogenates were plated on Middlebrook 7H11 agar supplemented with 10% Middlebrook OADC plus MGIT PANTA antibiotic cocktail (BD) to prevent contamination with other bacteria. Homogenates were prepared from single lung-draining mediastinal lymph nodes and either both lungs or the right lung only, as indicated in the relevant figures. Plates were incubated for 3 wk at 37°C before enumerating colonies and determining CFUs per organ.

### Organ single-cell suspensions

Lungs were lightly perfused by injection of PBS via the right ventricle of the heart, prior to dissecting out organs. In some experiments, single-cell suspensions were prepared from three lobes of the right lung, rather than all five lung lobes. These data are reported as “per lung prep,” rather than “per pair lungs” in the relevant figures. Lungs and lung-draining mediastinal lymph nodes were cut into small pieces with scissors and collected into serum-free RPMI, supplemented with 5% penicillin and streptomycin. Tissue was incubated in 1 ml total volume with 25 μg/ml DNase I (Sigma-Aldrich) and either 250 μg/ml (lung) or 48 μg/ml (lymph node) Liberase TM (Sigma-Aldrich) in serum-free RPMI at 37°C for 30 min with regular agitation. EDTA in PBS was added to a final concentration of 9.1 mM to stop digestion, and samples were placed on ice. Tissue was gently pressed through 70-μm strainers and flushed with RPMI supplemented with 10% fetal bovine serum and 5% penicillin and streptomycin. For some experiments, BAL was obtained by flushing lungs three times with 1 ml of PBS via the trachea using blunted 19G needles secured with suture thread. Red blood cells were lysed in all samples by suspending pellets in ammonium chloride lysis buffer for 4 min at room temperature before resuspending in supplemented RPMI. All samples were counted using a hemocytometer and Trypan blue exclusion to determine live and total cell numbers.

### Flow cytometry

Cells were washed in PBS before staining with Fixable Live/Dead Blue dye (Thermo Fisher Scientific, HN878 experiments) or Zombie Aqua dye (6C4 and 4I2 experiments; BioLegend). Cells were stained with combinations of the following fluorophore-conjugated antibodies, in PBS supplemented with 2% fetal bovine serum and 1 mM EDTA, in the presence of anti-mouse CD16/CD32 antibody (clone 2.4G2, Harlan for HN878 experiments; clone 93; BioLegend for 6C4 and 4I2 experiments) to block nonspecific binding to Fc receptors: CD11b-FITC (clone M1/70; BioLegend), XCR1-PERCP/Cy5.5 (clone ZET; BioLegend), Siglec F-Brilliant Violet 421 (clone E50-2440; BD), Ly6C-Brilliant Violet 510 (clone HK1.4; BioLegend), CX_3_CR1-Brilliant Violet 650 (clone SA011F11; BioLegend), MerTK-Brilliant Violet 711 (clone 108928; BD), I-A/I-E-Brilliant Violet 785 (clone M5/114.15.2; BioLegend), CD64-PE (clone X54-5-7.1; BioLegend), Ly6G-PE/Dazzle 595 (clone 1A8; BioLegend), CD26-PE/Cy7 (clone H194-112; BioLegend), CD5-APC (53-7.3; BioLegend), NKp46-APC (29A1.4; BioLegend), TER-119- APC (clone TER-119; BioLegend), CD19-APC (clone 6D5; BioLegend), Thy1.2-APC (clone 53-2.1; BioLegend), CD3ε-APC (clone 145-2C11; BioLegend), CD11c-APC/Cy7 (clone N418; BioLegend), Ly6G-APC/Cy7 (clone 1A8; BioLegend), CD3ε-APC/Cy7 (clone 145-2C11; BioLegend), CD19-APC/Cy7 (clone 6D9; BioLegend), Siglec F-Brilliant Violet 786 (clone E50-2440; BD), CD11c-PE/Cy7 (clone N418; BioLegend), I-A/I-E-Brilliant Violet 650 (clone M5/114.15.2; BioLegend), Ly6C-Brilliant Violet 421 (clone HK1.4; BioLegend), Ly6G-APC/Cy7 (clone 1A8; BioLegend), F4/80-Alexa Fluor 488 (clone BM8; BioLegend), TER-119- Alexa Fluor 488 (clone TER-119; BioLegend), Siglec F- Alexa Fluor 488 (clone E50-2440; BD), CD19-PERCP/Cy5.5 (clone 6D5; BioLegend), CD3ε-Brilliant Violet 421 (clone 145-2C11; BioLegend), CD4-Brilliant Violet 650 (clone RM4-5; BioLegend), CD103-Brilliant Violet 711 (clone 2E7; BioLegend), CD62L-Brilliant Violet 785 (clone MEL-14; BioLegend), GL7-PE (clone GL7; BioLegend), NKp46-PE/Dazzle 594 (clone 29A1.4; BioLegend), CD38/PE/Cy7 (clone 90; BioLegend), CD69-APC (clone H1.2F3; BioLegend), CD45-Alexa Fluor 700 (clone 30-F11; BioLegend), CD44-APC/Cy7 (clone IM7; BioLegend), CD8α-Brilliant UV 737 (clone 53-6.7; BD), CD103-APC (clone 2E7; BioLegend), CD62L-PE (clone MEL-14; BioLegend), CD69-Brilliant Violet 650 (clone H1.2F3, BioLegend), CD8α-Brilliant Violet 605 (clone 53-6.7; BD), and CD4-Brilliant Violet 785 (clone GK1.5; BioLegend).

Data were acquired on either an X20 (HN878 experiments) or LSR Fortessa (6C4 and 4I2 experiments) flow cytometer (both BD). Data were analyzed using FlowJo v10 (BD) using hierarchical gating, as shown in Fig. S1.

### scRNA-seq

Lung cells in single-cell suspension were enriched for CD45^+^ leukocytes using the magnetic bead–based mouse CD45 positive selection kit from Stem Cell Technologies as per the manufacturer’s instructions, which yielded ≥70% live, CD45.2^+^ cells per sample. Cells were fixed using the Fixation Kit from 10X Genomics as the per manufacturer’s instructions, made up to a final concentration of 4% formaldehyde with molecular biology grade formaldehyde (Sigma-Aldrich). Cells were fixed for 22 h at 4°C before quenching as the per manufacturer’s instructions and storing, supplemented with molecular biology grade glycerol (Sigma-Aldrich, 10% final concentration), at −80°C until analysis.

Cells were thawed and hybridized for 22 h to probes from the Mouse 16-Plex Fixed RNA Profiling kit (10X Genomics), as per the manufacturer’s instructions. All samples were split in two and hybridized to two different barcoded probe sets to increase cell recovery, except for two out of three uninfected control samples from each mouse strain. This resulted in a total of 18 biological samples run across two 16-Plex pools. Cells from C57BL/6 and C3HeB/FeJ mice were combined in equal numbers per barcode into individual pools, which were washed, concentrated, and loaded on 10x chip Q, where the fixed and probe-hybridized single-cell suspensions were partitioned into nanoliter-scale Gel Beads-in-Emulsion (GEMs). A pool of ∼737,000 10x GEM Barcodes was sampled separately to index the contents of each partition. Inside the GEMs, probes were ligated, and the 10x GEM Barcode was added. Barcoded and ligated probes were then pre-amplified in bulk, after which gene expression libraries were generated and sequenced on a NovaSeq 6000 instrument (Illumina) using sequencing read configuration: 28-10-10-90. Raw and processed scRNA-seq data are deposited in the GEO at accession GSE298787.

### scRNA-seq data processing and analysis

Cell Ranger v7.0.1 filtered matrices, aligned to the mm10 2020-A mouse genome (official 10X mouse pre-built reference), were processed in R v4.3.2 using Seurat v4.4.0 (with SeuratObject v4.1.4). DecontX (Yang et al., 2020) (R package celda v1.18.2) was used to model ambient RNA contamination and remove ambient RNA. Low-quality cells with <1,000 unique molecular identifier counts or <500 unique genes after ambient RNA removal, or with >5% mitochondrial genes, were filtered out. Scrublet (Wolock et al., 2019) was used to mark and remove likely doublet cells.

Integration, clustering, and marker gene identification were implemented using the Seurat functions FindIntegrationAnchors(), IntegrateData(), FindClusters(), and FindAllMarkers(), using the top 2,000 variable features, the first 40 principal components, resolution of 0.3 to give a total of 31 clusters, and the robust principal component analysis method of integration. Five minor clusters were manually removed from integrated data due to (1) containing poorly defined cells with high similarity to cells removed following DecontX clean-up (one cluster, 2165 cells), (2) containing residual doublets due to expression of incompatible marker genes of distinct cell lineages (three clusters, <200 cells total), and (3) containing only two cells. A final annotation was assigned to the resultant 26 clusters, based on manual inspection of marker gene lists and a cluster-level annotation assigned by R package clustifyr v1.5.1 (Fu et al., 2020) using the Mouse Cell Atlas (Han et al., 2018), Tabula Muris 10X and SmartSeq2 (Schaum et al., 2018), and ImmGen (Gautier et al., 2012; Heng et al., 2008) as reference datasets. Six clusters were determined to represent low numbers of contaminant epithelial, endothelial, and stromal cells (grey clusters in Fig. 3 a) and were not analyzed further.

### Differential abundance analysis of scRNA-seq data

A Dirichlet-multinomial regression model was used to test for changes in cell abundance between conditions to account for the compositional nature of cell count data. This regression model was calculated using the DirichReg() function from the DirichletReg R package (Maier, 2014). When statistically testing for differences in cell abundance between mouse strains at specific time points, a generalized linear model was fit using the quasi-likelihood F test to account for overdispersion between models, implemented using the glmQLFit() function within the edgeR R package (Chen et al., 2024, *Preprint*).

### Inference of cell-to-cell communication from scRNA-seq data

Cell-to-cell interactions were inferred using the R package CellChat v1.1.3, with default parameters (Jin et al., 2021). The “population.size” parameter was set to TRUE when computing the inferred interaction between cell subsets. Relative predicted signaling contribution of different ligand–receptor pairs and cell types was quantified as indicated in the relevant figures.

### Gene signature evaluation in scRNA-seq clusters

Expression of a published in vitro–derived type I IFN–response signature (Kotov et al., 2023) was scored in cells using the Seurat function AddModuleScore().

### Bulk RNA extraction and RNA-seq

Lung tissue was homogenized in TRI reagent using a FastPrep-24 homogenizer and Lysing Matrix D tubes (MP Biomedicals) before centrifuging at 10,000 × *g* for 10 min at 4°C to pellet debris. Whole lung sets (five lobes) were used for comparisons between C57BL/6 and C3HeB/FeJ mice. Bottom right lung lobes were used for the anti-IFNAR experiment in C3HeB/FeJ mice described in Fig. 10. This was previously unused tissue from experiments reported in our previous work (Moreira-Teixeira et al., 2020a). RNA was extracted from lung homogenates using the DirectZol Mini Kit with on-column DNase I digestion (Zymo), as per the manufacturer’s instructions. BAL cell pellets were resuspended in TRI reagent and vortexed at high speed for ≥10 s to lyse. RNA was extracted from BAL lysates using the DirectZol Mini Kit with on-column DNase I digestion (Zymo), as per the manufacturer’s instructions. RNA integrity of all samples was determined to range from 5.7–9.4 (median 7.9).

All bulk RNA-seq libraries were prepared using total RNA stranded library preparation kits as appropriate for input RNA quantity. RNA-seq libraries from whole lungs of HN878-infected C57BL/6 and C3HeB/FeJ mice were prepared using the KAPA total RNA library preparation kit with RiboErase (Illumina). RNA-seq libraries from whole BAL of HN878-infected C57BL/6 and C3HeB/FeJ mice and from lung tissue samples obtained from anti-IFNAR-treated HN878-infected C3HeB/FeJ mice were prepared using the total RNA library prep kit with Polaris ribosomal RNA depletion (Watchmaker Genomics). Libraries were sequenced on an Illumina NovaSeq 6000 or NovaSeq X (for anti-IFNAR experiment) sequencer platform generating ∼25 million 100-bp paired end reads per sample. Raw and processed bulk RNA-seq data are deposited in the GEO at accession GSE298786.

### Bulk RNA-seq analysis

RNA-seq data were processed and aligned to the mm10 genome using the default nf-core/rnaseq pipeline v3.16.1, built with Nextflow. Briefly, this runs Trim Galore, STAR, and Salmon for trimming, alignment, and transcript count quantification, among additional quality controls. An in-depth description of the pipeline can be found at https://nf-co.re/rnaseq/3.16.1/.

Differential gene expression was performed using DESeq2 v1.42.1. Expression values were normalized by rlog transformation and Wald’s test implemented to test for differential expression, as compared with respective uninfected control samples. Genes were determined to be differentially expressed with a fold change of ≥1.5 and Benjamini–Hochberg-adjusted P value of <0.05. Total DEGs were clustered based on their normalized expression profile across all groups, using k-means clustering. Optimal k was determined by iterating through a k from 5 to 15 and manually selecting the number of clusters that best captured the observed expression pattern.

### Visualization of RNA-seq data

All data were visualized using ggplot2 in R. R packages scCustomize and ggh4x were used to customize Seurat native plotting functions for scRNA-seq data visualization.

### Lung tissue immunostaining

Lungs were lightly perfused and inflated in situ by injection of PBS via the right ventricle of the heart. Lung tissue was fixed in freshly prepared methanol-free 4% formaldehyde in PBS for 48 h before processing to wax blocks using a Tissue-Tek VIP 6 AI processor. All immunostaining was performed automatically using the BondRx (Leica Biosystems) using 3-μm formaldehyde-fixed, paraffin-embedded sections.

For S100A9 immunohistochemistry, sections were baked for 1 h at 60°C prior to automated staining with antibody clone 2B10 (The Francis Crick Institute Cell Services), using Protease 1 antigen retrieval, DISCOVERY OmniMap anti-Rat HRP, and DISCOVERY ChromoMap DAB Kit on a Ventana Discovery Ultra autostainer (all Roche). Bright-field imaging of whole slides was performed on the Axio Scan Z1 slide scanner (Zeiss).

For multiplex immunofluorescence staining, sections were baked for 1 h before blocking in 3% hydrogen peroxide, followed by 0.1% bovine serum albumin in PBS-Tween. Antigen retrieval and stripping was performed between each antibody incubation step by incubating with Epitope Retrieval Solution 1 (Leica Biosystems) at pH 9 for 20 min at 95°C. Antibodies for two staining panels were applied with Opal pairings (all Akoya Biosciences) in the orders listed here. For the macrophage, neutrophil, and T and B cell panel: biotinylated B220 (clone RA3-6B2; BD) with Opal 620, CD8α (clone EPR21769; Abcam) with Opal 690, Ly6G (clone E6Z1T; Cell Signaling Technology) with Opal 570, CD31 (clone EPR17259; Abcam) with Opal 480, CD4 (clone EPR19514; Abcam) with Opal 520, and CD68 (clone EPR23917-164; Abcam) with Opal 780. For the NET panel: myeloperoxidase (AF3667; R&D Bio-Techne) at 1:400 with Opal 570 at 1:300, CitH3 (ab5103; Abcam) at 1:500 with Opal 620 at 1:300, Ly6G (87048S; CST) at 1:100 with Opal 520 at 1:300, CD4 (ab183685; Abcam) at 1:750 with Opal 690 at 1:150, and CD68 (ab283654; Abcam) at 1:2,500 with Opal 780 at 1:100 & 1:25. Streptavidin-HRP (Agilent Technologies) was used as a secondary reagent for biotinylated B220 antibody; HRP Horse Anti-Goat IgG Polymer Detection Kit (MP-7405; Vector Laboratories) was used as secondary for the myeloperoxidase antibody, which was raised in goat; Bond Anti-Rabbit Polymer (Leica Biosystems) was used for the other antibodies, which were raised in rabbit. Slides were counterstained with DAPI and mounted with ProLong; Gold Antifade reagent (both Thermo Fisher Scientific). Slides were imaged using the PhenoImager HT slide scanner (Akoya Biosciences) at 20X using MOTiF scanning mode. Spectral unmixing and removal of tissue immunofluorescence for the macrophage, neutrophil, and T and B cell panels was performed using Phenochart 1.1.0 and InForm 2.6.0 software (Akoya Biosciences), and for the NET panel was performed using the integrated spectral unmixing feature within the PhenoImager HT 2.1 software.

### Analysis of multiplex immunofluorescence images

Exported.QPTIFF files from InForm 2.6.0 were imported into QuPath v0.5.1 software (Bankhead et al., 2017) and stitched digitally to generate whole tissue scans. Cell segmentation was performed based on the DAPI signal, using the Stardist extension for QuPath (Schmidt et al., 2018, *Preprint*), using parameters determined on representative training images from all infection time points in both C57BL/6 and C3HeB/FeJ mice. Lesions were identified as areas of dense CD68 macrophage staining, initially by training a random trees pixel classifier using the DAPI, CD68, and Ly6G channels, followed by manual adjustment of annotated lesions to exclude perivascular or peribronchiolar leukocyte aggregates and filtering of small cellular aggregates <12,000 μm^2^. All detected lesions were annotated in each section, either in whole lung (data in Figs. 5 and S3) or on left lungs (all other analysis). Cell detection for lymphocytes (CD4^+^, CD8α^+^, or B220^+^ cells) was performed in QuPath using a random trees object classifier trained on staining for CD4, CD8α, and B220 markers. As Ly6G staining was observed both in discrete cells and in myeloid aggregates in lesions, Ly6G staining was quantified by pixel classification, rather than performing cell segmentation and object classification, by training a random trees pixel classifier on the DAPI and Ly6G channels. Macrophage and NET annotations were identified by pixel classification using machine training methods based on DAPI, as well as CD68 and CitH3 signal, respectively. In some analyses, lesions were binned into those with low (<20%), intermediate (≥20% <40%), or high (≥40%) area coverage with Ly6G^+^ pixel classifications, or low (ratio <0.2), intermediate (ratio ≥0.2 <0.4), or high (ratio ≥0.4) CitH3^+^ relative to Ly6G^+^ pixel classifications. In some analyses, total CD4^+^ T cells across whole lung tissue were enumerated, including those in lesions, perivascular infiltrates, peribronchiolar infiltrates, and non-lesional lung parenchyma. In other analyses, frequency of CD4^+^ T cells in non-lesional lung parenchyma was quantified by determining the number of CD4^+^ T cells per μm^2^ across three to eight equal-sized regions per mouse lacking any signs of inflammation, for comparison to lesional tissue from the same mouse. Distance analysis of CD4^+^ T cells relative to macrophages in lesions was performed using the signed distance to annotation tool in QuPath to calculate the distance of CD4^+^ T cells to the edge of the nearest CD68^+^ macrophage annotation.

### Visualization and statistical analysis for non-transcriptomic data

Statistical tests selected were appropriate for the number of groups compared, variables assessed, and distribution of data, as indicated in the relevant figure legends. Aligned rank transform analysis for nonparametric two-way ANOVA analysis and downstream post hoc analysis were performed when required using the ARTool R package (Elkin et al., 2021; Wobbrock et al., 2011). Comparisons of inter-related lesion composition parameters between groups were performed using the DirichReg() function from the DirichletReg R package (Maier, 2014). All other statistical analyses and data graphing were performed in Prism v10 (GraphPad).

### Online supplemental material

Fig. S1 shows the additional bacterial CFU, flow cytometry, and scRNA-seq data in support of Figs. 1, 2, and 3. Fig. S2 shows the additional bulk and scRNA-seq analysis in support of Fig. 4. Fig. S3 shows the additional lung multiplex immunofluorescence images and analysis in support of Figs. 5 and 6. Fig. S4 shows the additional scRNA-seq analysis in support of Fig. 7. Fig. S5 shows the additional bulk and scRNA-seq analysis and lung multiplex immunofluorescence images and analysis in support of Figs. 7, 9, and 10. Data S1 contains the additional results of CellChat pathway analysis of scRNA-seq data from lung leukocytes from *M. tuberculosis* HN878–infected C57BL/6 and C3HeB/FeJ mice.

## Supplementary Material

Data S1 shows the predicted early ligand–receptor interactions in lungs of C57BL/6 and C3HeB/FeJ mice.

## Data Availability

Raw and processed RNA-seq data shown throughout the figures are deposited in the GEO at accessions GSE298786 (bulk RNA-seq) and GSE298787 (scRNA-seq). All code for the analysis from raw data to final figures is available at https://github.com/ogarralab/workflowr, with the specific release available through Zenodo https://doi.org/10.5281/zenodo.16894788. The bulk and scRNA-seq data generated in this work are also available for access in an interactive and accessible format via a web app: https://ogarra.shinyapps.io/earlymousetb/.
