# Supplementary material for: Type I IFN drives neutrophil swarming, impeding lung T cell–macrophage interactions and TB control
Source: J Exp Med. 2025 Sep 23;222(12):e20250466. doi: 10.1084/jem.20250466 (PMC12456410; doi:10.1084/jem.20250466)
Supplement: Data S1 — shows the predicted early ligand–receptor interactions in lungs of C57BL/6 and C3HeB/FeJ mice. [file jem_20250466_datas1.pdf]

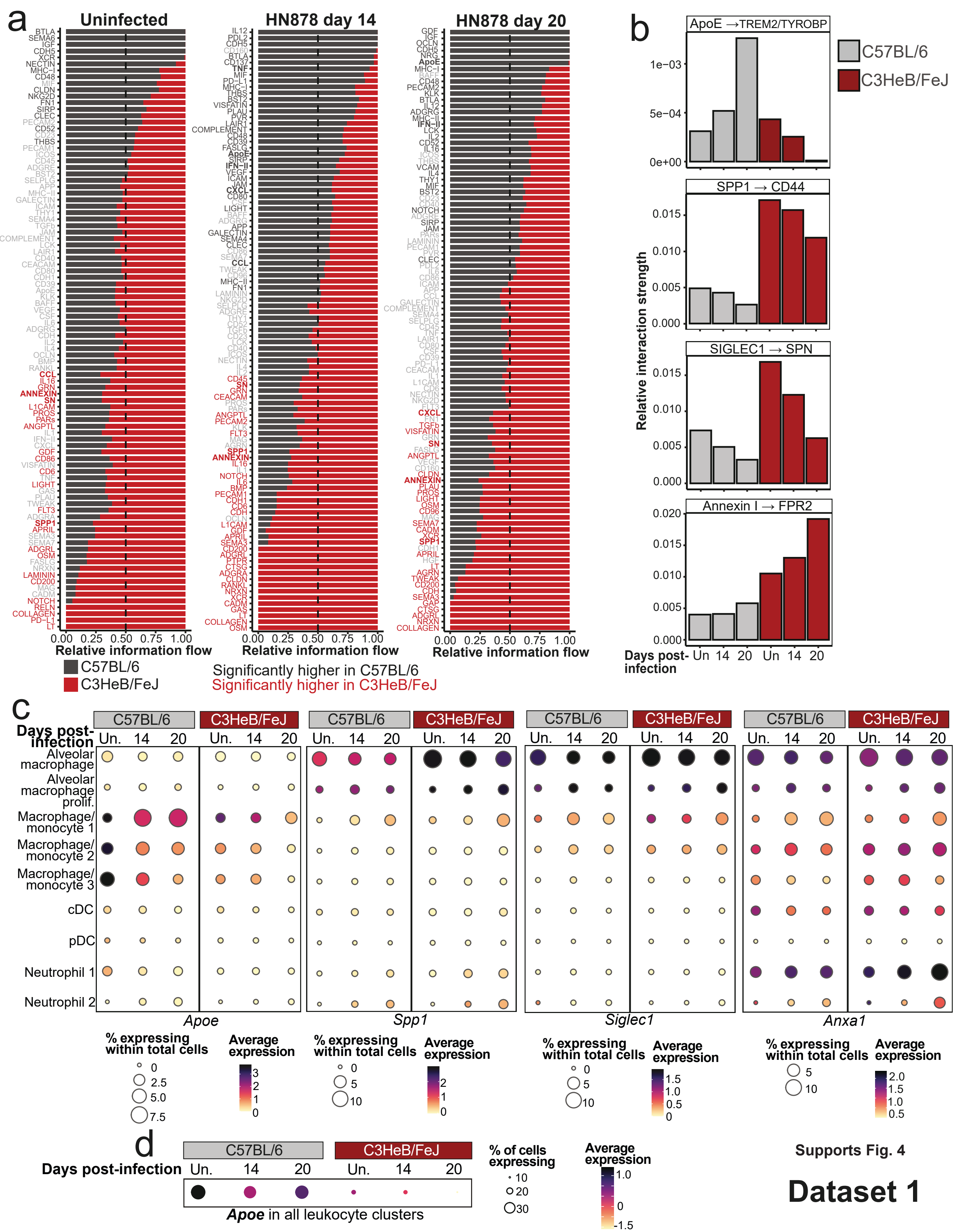

Supports Fig. 4

Dataset 1

Data S1. **Predicted early ligand-receptor interactions in lungs of C57BL/6 and C3HeB/FeJ mice.** **(a)** Relative CellChat predicted interaction activity of pathways in C57BL/6 compared to C3HeB/FeJ mice under each condition, based on scRNA-seq data. Greyed-out pathway names indicate no statistically significant difference. Pathways discussed in further detail are shown in bold. **(b)** Bar plots showing the relative contribution of the indicated receptor/ligand interactions to total inferred interaction activity in each group. **(c and d)** Dot plots showing expression of the indicated ligand genes in myeloid cell populations or within total cells in scRNA-seq data. Circle sizes represent the abundance of cells expressing the gene, as a percentage of total cells. Circle color is proportional to the mean expression of the gene within all cells in the cluster. Data shown are from a single scRNA-seq experiment and plots show combined data from cells from N=3 mice per group.
